# Phytoplasma Effector SJP8 Suppresses Host Immunity by Promoting the Degradation of ZjMYB15 and ZjMYB86‐like to Perturb Jasmonic Acid and Hydrogen Peroxide Homeostasis in Jujube

**DOI:** 10.1111/mpp.70315

**Published:** 2026-07-10

**Authors:** Ying Yang, Haizhen Nie, Weikai Chen, Yaru Xu, Bo Wu, Ling Ma, Zhonghua Liu, Xiaoming Pang

**Affiliations:** ^1^ State Key Laboratory of Tree Genetics and Breeding, National Engineering Research Center of Tree Breeding and Ecological Restoration, Key Laboratory of Genetics and Breeding in Forest Trees and Ornamental Plants, Ministry of Education, College of Biological Sciences and Biotechnology Beijing Forestry University Beijing China

**Keywords:** host immunity, hydrogen peroxide, jasmonic acid, SJP8, ZjMYB15, ZjMYB86‐like

## Abstract

‘*Candidatus* Phytoplasma ziziphi’ infection causes severe damage to jujube ( *Ziziphus jujuba* ), inducing symptoms such as branch clustering, dwarfism, chlorosis and phyllody, leading to significant economic losses. However, the pathogenic mechanisms underlying jujube witches'‐broom (JWB) disease remain largely unknown. In this study, we identified SJP8 as a key secreted effector protein of ‘*Ca*. P. ziziphi’. Overexpression of *SJP8* in jujube recapitulated the characteristic JWB‐like symptoms, including dwarfism and leaf chlorosis. Transcriptome analysis revealed that SJP8 suppressed host immunity by downregulating jasmonic acid (JA)‐related genes and upregulating hydrogen peroxide (H_2_O_2_)‐related genes. SJP8 interacts with ZjMYB15 and ZjMYB86‐like and promotes their degradation via the 26S proteasome pathway. Genetic transformation experiments demonstrated that ZjMYB15 and ZjMYB86‐like positively regulate plant height and the expression of JA‐related genes while repressing H_2_O_2_‐associated genes. ZjMYB15 and ZjMYB86‐like bind to their cognate motifs to activate *ZjJAIPHX1* (encoding a *jasmonate‐induced protein homologue isoform X1*) and *ZjPOD43* (encoding *peroxidase 43*) transcription, thereby increasing JA content and decreasing H_2_O_2_ levels. Collectively, these results establish that the SJP8‐ZjMYB15/ZjMYB86‐like‐*ZjJAIPHX1*/*ZjPOD43* module suppresses plant immunity by perturbing JA and H_2_O_2_ homeostasis, thus both elucidating a phytoplasma effector's virulence strategy and providing molecular targets for breeding resistant jujube.

## Graphical abstract

SJP8 suppresses plant immunity by perturbing JA and H_2_O_2_ homeostasis via the SJP8‐ZjMYB15/ZjMYB86‐like‐*ZjJAIPHX1*/*ZjPOD43* module, leading to dwarfism and chlorosis.

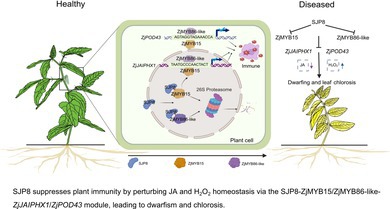

## 1 Introduction

Jujube ( *Ziziphus jujuba* ) is a perennial fruit tree of great economic and ornamental value, cultivated in Asia, Africa and parts of the Americas for over 7000 years (Liu et al. 2020; Guo et al. 2021). However, the jujube industry has recently suffered severe losses due to jujube witches'‐broom (JWB) disease, a lethal disease caused by ‘*Candidatus* Phytoplasma ziziphi’ (Jung et al. 2003; Wang, Song, et al. 2018). Phytoplasmas are obligate plant and insect symbionts that typically require hosts for dispersal (Lee et al. 2000; Bertaccini 2007; Hogenhout et al. 2008; MacLean et al. 2011; Wang et al. 2024). Symptoms of phytoplasma infection include the proliferation of branches, leaf chlorosis, conversion of flowers into leaf‐like structures, and dwarfing of plants (Hoshi et al. 2009; Sugio et al. 2011; Orlovskis and Hogenhout 2016; Huang et al. 2021). JWB disease is known as the ‘cancer’ of jujube trees, underscoring the urgent need to elucidate its pathogenic mechanisms.

Phytoplasmas render plants susceptible to disease by secreting effector proteins into the host, thereby initiating a complex plant–pathogen interaction. SAP54_AY‐WB_ from the ‘*Candidatus* Phytoplasma asteris’ strain aster yellows witches' broom (AY‐WB) induces phyllody in *Arabidopsis thaliana* by degrading MIKC‐type MADS‐box transcription factors (MacLean et al. 2011, 2014). SAP05_AY‐WB_ induces witches' broom‐like leaf and shoot proliferations by degrading AtSPL and AtGATA family proteins in a ubiquitination‐independent manner (Huang et al. 2021). Overexpression of SAP06_AY‐WB_ leads to impaired vegetative growth and enhanced seed dormancy in transgenic *A. thaliana* (Correa Marrero et al. 2024). SWP12_WBD_ from wheat blue dwarf (WBD) phytoplasma degrades TaWRKY74 via the 26S proteasome to suppress host resistance, thereby causing yellowing in wheat (Bai et al. 2022). Collectively, these studies reveal that phytoplasma effectors manipulate plant development and immunity by targeting key host regulators, providing insights for breeding disease‐resistant plants.

Plants possess two layers of immunity: pathogen‐associated molecular pattern (PAMP)‐triggered immunity (PTI) activated by cell‐surface receptors, and effector‐triggered immunity (ETI) mediated by resistance proteins. Pathogen effectors can suppress PTI, leading to infection (Akira et al. 2006; Houterman et al. 2008). Both immunity types enhance reactive oxygen species (ROS) production, callose deposition and hormone signalling (Dodds and Rathjen 2010; Tsuda and Katagiri 2010). ROS generation is an early signalling event in plant defence (Wang et al. 2022). Hydrogen peroxide (H_2_O_2_), a major form of ROS, participates in host defence during plant–pathogen interactions (Li et al. 2014; Waszczak et al. 2018; Tondo et al. 2020). Its main sources are chloroplasts, mitochondria and peroxisomes (Saxena et al. 2016). H_2_O_2_ production is tightly regulated by class III peroxidases (PODs), which can act as producers or scavengers depending on the cycle (Torres 2010; Aviello and Knaus 2018). Exogenous arachidonic acid (ARA) enhances tomato defence enzymes (polyphenol oxidase, peroxidase, catalase, phenylalanine ammonia‐lyase, ascorbate peroxidase) and activates the phenylpropanoid pathway (upregulating *4CL*, *PAL*, *POD*, *CCoAOMT*, *CYP98A3*), which together with MAPK signalling improve resistance to *Botrytis cinerea* (Gao et al. 2026). In JWB‐infected jujube, *ZjPOD51* overexpression upregulates defence genes (e.g., *ZjRbohD*) and promotes lignin accumulation via ZjMYB44, enhancing phytoplasma resistance (Zhang, Li, Wei, et al. 2025).

Plant defence against pathogens and insects is regulated through a complex network of cross‐communication signalling pathways involving hormones such as salicylic acid (SA), jasmonic acid (JA), ethylene (ET), brassinosteroids (BRs), abscisic acid (ABA) and gibberellins (GAs) (Spoel et al. 2003; Aerts et al. 2021; Yan et al. 2024). JA and its derivatives, collectively referred to as jasmonates, regulate plant growth, development, secondary metabolism, defence against insect herbivory and pathogen infection (Wasternack and Song 2017). For example, overexpression of the TENGU_OY‐M_ effector from onion yellows phytoplasma strain M (OY‐M) in *A. thaliana* inhibits the transcription of auxin response factors (AtARF6 and AtARF8) and downregulates JA content, leading to a certain degree of sterility (Minato et al. 2014). The JA pathway mainly comprises biosynthesis, metabolism and signal transduction, which collectively participate in plant growth, development and immune responses (Guo et al. 2020; Gupta et al. 2020; Roychowdhury et al. 2025). In transgenic *A. thaliana* , SAP11_AY‐WB_ primarily targets and destabilises CYC/TB1 BRC1 (AtTCP18) transcription factors to induce witches' broom symptoms and suppress JA defences in *A. thaliana* (Bai et al. 2009; Sugio et al. 2011, 2014). Thus, JA plays a pivotal role in the tripartite interactions among plants, pathogens and insect vectors.

The MYB protein family features a highly conserved DNA‐binding domain and includes both positive and negative regulators of stress responses (Buscaill and Rivas 2014; Jiang and Rao 2020). MYB transcription factors are known to regulate phenylpropanoid biosynthesis, lignin accumulation and H_2_O_2_ production, thereby linking them to both growth and defence (Pratyusha and Sarada 2022). For instance, overexpression of *OsMYB14* reduces rice plant height by modulating GA and auxin pathways (Kim et al. 2024). *AtMYB15* overexpression increases resistance to *Pseudomonas* by increasing lignin content, whereas its deletion reduces both lignin content and resistance (Chezem et al. 2017). AtMYB61 plays pleiotropic roles in mucilage production, stomatal regulation, xylem development and lateral root formation (Penfield et al. 2001; Liang et al. 2005; Romano et al. 2012). OsMYB55/61 confers JA‐mediated resistance to *Xanthomonas oryzae* pv. *oryzae* by upregulating *OsPOD26* and promoting lignin production (Uji et al. 2024). However, the roles of MYB transcription factors in jujube stress responses remain underexplored due to the immature transformation system and long growth cycle.

Previous studies on the pathogenic mechanisms of JWB disease have revealed that two SAP11_AY‐WB_ homologues, SJP1_JWB_ and SJP2_JWB_, interact with several host transcription factors—including ZjTCP2, ZjTCP7 and ZjBRC1—inducing morphological alterations in branch and leaf architecture (Zhou et al. 2021; Ma, Zheng, et al. 2024; Ma, Huang, et al. 2024). Moreover, CRISPR‐induced *zjtcp7* jujube mutants exhibit a shoot proliferation phenotype (Chen et al. 2022). Similarly, the SJP3_JWB_ effector, a functional analog of SAP54_AY‐WB_, disrupts *ZjSVP3* expression—a regulator of floral organ identity and flowering timing—leading to symptoms such as clustering and leaflet formation in infected *A. thaliana* (Deng et al. 2024). SJP8 (locus AYJ01076.1) was initially reported by Deng et al. (2021) and later designated as Zaofeng1 by Chen et al. (2022), though neither study elucidated its biological function (Deng et al. 2021; Chen et al. 2022). Recently, Wan et al. (2025) identified SJP8 (also referred to as JWB790 in their study) as a key virulence effector in JWB phytoplasma. They demonstrated that its expression in model plants suppresses immunity and facilitates pathogen colonisation (Wan et al. 2025). However, the molecular host targets of SJP8 and the specific signalling pathways it intercepts to cause disease symptoms in its natural host, jujube, remain unexplored.

In this study, we further characterise SJP8 as a key effector that induces dwarfism and chlorosis in jujube, thereby advancing our mechanistic understanding of its pathogenicity. We demonstrate that SJP8 interacts with and promotes the degradation of ZjMYB15 and ZjMYB86‐like proteins, which act as positive regulators of the transcription of *jasmonate‐induced protein homologue isoform X1* (*ZjJAIPHX1*) and *peroxidase 43* (*ZjPOD43*). This interference disrupts the homeostasis of JA and H_2_O_2_, ultimately suppressing host immunity. Collectively, our findings provide novel molecular insights into JWB disease pathogenesis and offer a theoretical foundation for future resistance breeding in jujube.

## 2 Results

### 2.1 SJP8 Is a Key Effector Protein of ‘*Ca*. P. ziziphi’ That Can Aggravate Viral Infection

To explore the pathogenic mechanisms of JWB, we identified 70 candidate effectors using the first published JWB phytoplasma genome (GenBank: GCA_003640545.1) (Figure S1a, Table S1; Wang, Song, et al. 2018). Together with transcriptome data from infected jujube and leafhoppers (NCBI: PRJNA1158699), we selected 15 effectors that exhibited higher expression levels in jujube than in leafhoppers (Figure S1b). Bioinformatics analysis revealed that SJP8 and SJP72 are likely unique to the JWB phytoplasma. Particularly, SJP8 is located within PMU5, a genomic region associated with pathogenicity and evolutionary variation in phytoplasmas (Chen et al. 2022). Despite a second JWB genome (GenBank: GCA_022058185.1) showing a different PMU arrangement, SJP8 is highly conserved between the two strains. Given its specificity and high expression in jujube, SJP8 was selected for further functional characterisation (Figure S1c,d).

**FIGURE 1:**
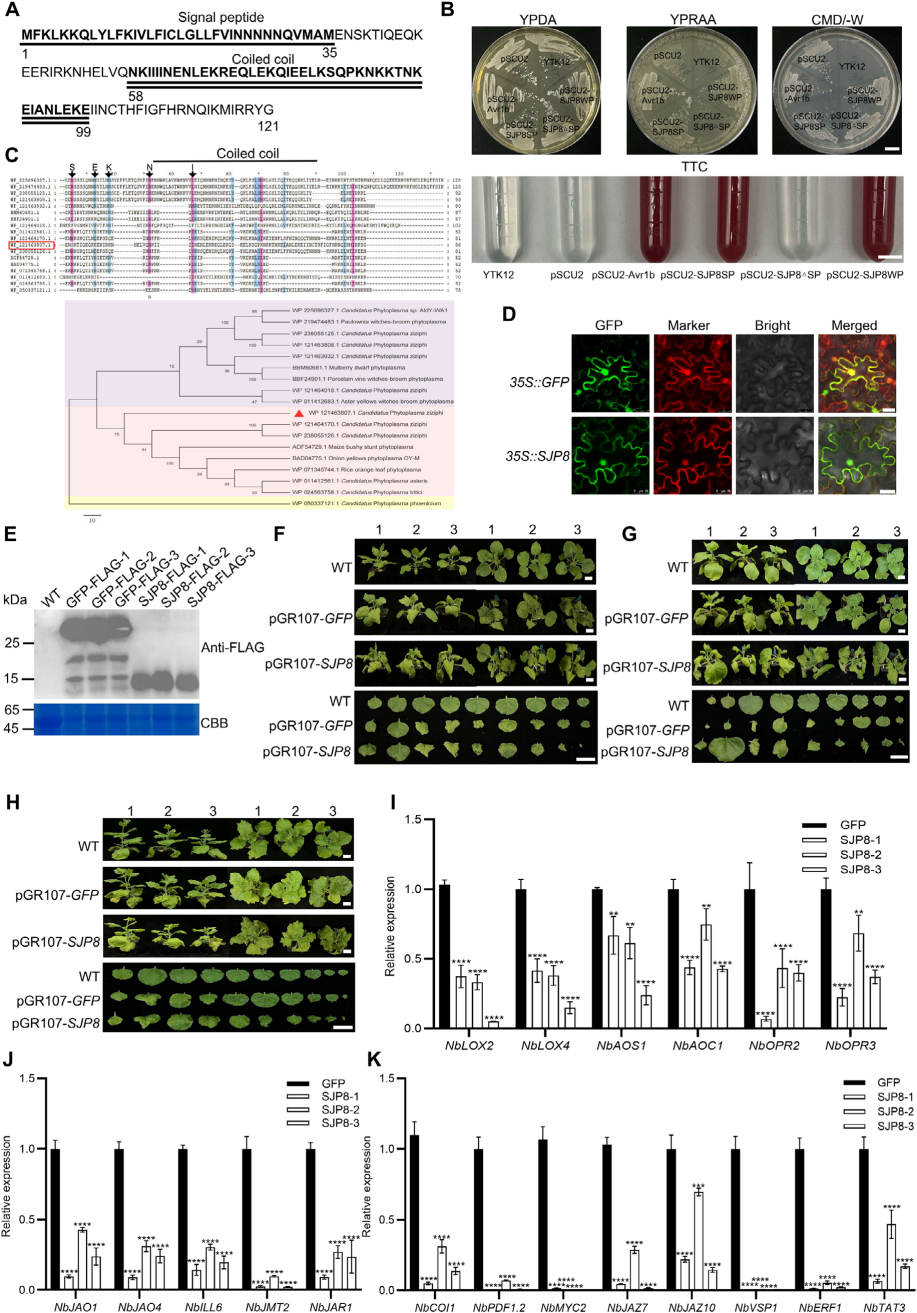
SJP8 is a key effector protein of ‘*Candidatus* Phytoplasma ziziphi’ that aggravates infection. (A) Amino acid sequence of SJP8. The single and double black lines indicate signal peptide and coiled‐coil domain, respectively. (B) Functional validation of the predicted signal peptide of SJP8. pSCU2‐*Avr1b* (*Avr1b* serves as a signal peptide) was used as a positive control; the empty pSCU2 vector served as a negative control. Scale bar = 1 cm. (C) Multiple sequence alignment and phylogenetic analysis of SJP8. Multiple sequence alignment of SJP8 with 11 phytoplasma protein sequences was performed using ClustalW in MEGA 11. The NCBI accession number (WP_121463807.1) of SJP8 is highlighted in a red box, and the coiled coil sequence is indicated by a black line. Conserved amino acid residues S, E, K, N and I are marked with black arrows. The phylogenetic tree illustrating the evolutionary relationship of SJP8 with 11 phytoplasma species was constructed using the neighbour‐joining method, and the SJP8 accession number (WP_121463807) is marked with a red triangle. Scale bar = 10. (D) Subcellular localisation of SJP8. GFP was used as a marker to assess SJP8 localisation; an empty GFP vector served as a control. The pBI121‐*OsGRX20*‐mCherry construct (mCherry fluorescence) served as a nuclear‐cytoplasmic co‐localisation marker, as OsGRX20 has been reported to localise to both the nucleus and cytoplasm (Ning et al. 2018). Scale bar = 25 μm. (E) Western blot analysis of SJP8 expression in *Nicotiana benthamiana* at 7 days post‐infiltration (dpi). The large subunit of ribulose‐1,5‐bisphosphate carboxylase/oxygenase (RuBisCO), visualised by Coomassie brilliant blue (CBB) staining, served as a loading control. Molecular weight markers (kDa) are indicated on the left. (F–H) Phenotypic observation of *N. benthamiana* at 7 dpi (F), 14 dpi (G) and 21 dpi (H). Numbers 1, 2 and 3 represent three biological replicates for wild‐type (WT), pGR107‐GFP and pGR107‐SJP8 lines. Scale bar = 5 cm. (I–K) Relative expression levels of jasmonic acid (JA) biosynthesis (I), metabolism (J) and signal transduction (K) genes in *N. benthamiana* leaves transiently overexpressing *SJP8* at 7 dpi, determined by reverse transcription‐quantitative PCR. *NbActin* was used as an internal reference gene. For panels (I–K), statistical analysis was performed using one‐way ANOVA with Tukey's test. Error bars represent the standard deviation (SD) of three technical replicates. Significance is indicated as **p < 0.01, ***p < 0.001, ****p < 0.0001. All experiments were repeated three times with consistent results.

Using SignalP‐6.0, SJP8 was predicted to be a 121‐amino acid protein with an N‐terminal signal peptide. SMART domain analysis identified a 42‐amino acid coiled‐coil (CC) domain (Figure 1A). A yeast secretion assay showed that TTC (2,3,5‐triphenyltetrazolium chloride) was reduced to a red insoluble product in strains expressing SJP8WP or SJP8SP, confirming the secretory function of the signal peptide (Figure 1B). BLASTp searches against the NCBI nr database identified 18 effector homologues from 11 phytoplasma species. SJP8 shared the highest identity (72.50%) with two ‘*Ca*. Phytoplasma ziziphi’ homologues, followed by moderate identities (53.45%–60.00%) with effectors from rice orange leaf phytoplasma, maise bushy stunt phytoplasma, onion yellows phytoplasma OY‐M, ‘*Ca*. Phytoplasma asteris’ and ‘*Ca*. Phytoplasma tritici’, with an overall species similarity of 38.53% (Figure 1C). Moreover, multiple sequence alignment identified partially conserved residues S, E, K, N and I at positions 38, 46, 51, 58 and 63, respectively, with three homologues exhibiting substitutions. Furthermore, subcellular localisation in *Nicotiana benthamiana* revealed that SJP8 localises to both the nucleus and the cytoplasm, a finding corroborated by plasmolysis assays (Figure 1D, Figure S2).

To investigate the pathogenicity of SJP8, we transiently overexpressed *SJP8* in *N. benthamiana*. At 7 days post‐infiltration (dpi), *SJP8*‐expressing leaves exhibited curling, crumpling and chlorosis, with symptoms progressively intensifying from 7 to 21 dpi (Figure 1E–H). Reverse transcription‐quantitative PCR (RT‐qPCR) analysis of leaves at 7 dpi showed that genes involved in JA biosynthesis, metabolism and signal transduction were significantly downregulated, whereas those associated with H_2_O_2_ production, scavenging and signal transduction were markedly upregulated (Figure 1I–K, Figure S3a–c). Collectively, these results indicate that SJP8 is a key effector of the JWB phytoplasma, probably compromising plant immunity by modulating the JA and H_2_O_2_ pathways.

### 2.2 *SJP8* Overexpression Induces Dwarfing and Leaf Chlorosis in Transgenic Jingzao 39

**FIGURE 2:**
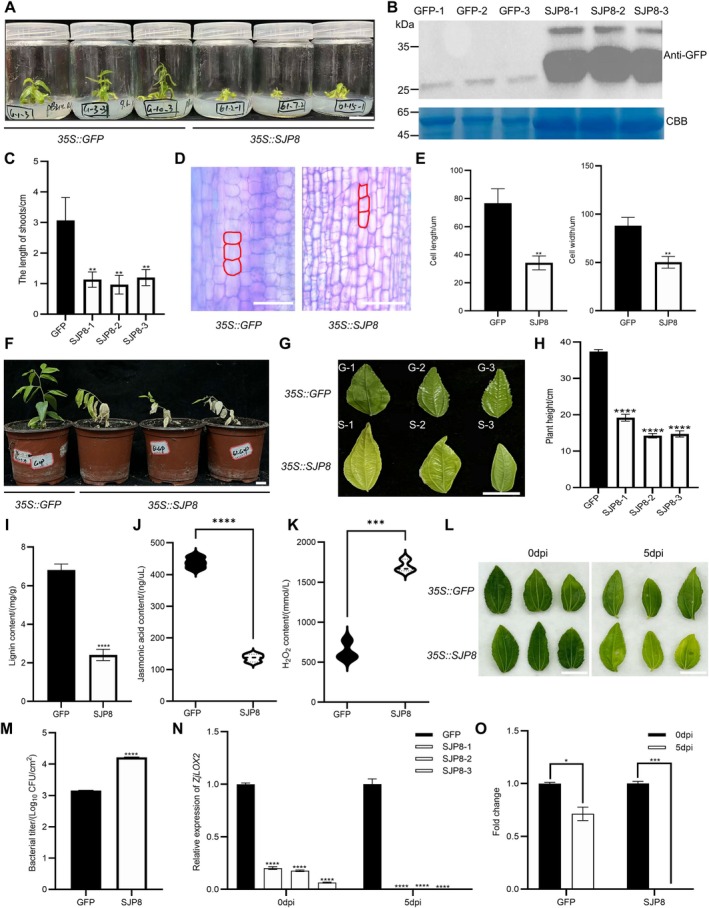
*SJP8* overexpression induces dwarfism and leaf chlorosis in transgenic Jingzao 39. (A) Phenotypes of various transgenic lines after 1 month of tissue culture. (B) Western blot analysis of SJP8 protein levels in the transgenic lines. The large subunit of ribulose‐1,5‐bisphosphate carboxylase/oxygenase (RuBosCO), visualised by Coomassie brilliant blue (CBB) staining, served as a loading control. Molecular weight markers (kDa) are indicated on the left. (C) Quantification of shoot length in different transgenic lines. (D) Cross‐sections of transgenic stems stained with methylene blue. Scale bar = 100 μm. (E) Quantification of cell length and width from stem cross‐sections shown in (D). (F) Representative image of transgenic plants 1 month after transplantation. Scale bar = 1 cm. (G) Phenotypes of apical leaves from transgenic plants 1 month after transplantation. Three independent empty vector control lines (G‐1, G‐2, G‐3) and *SJP8*‐overexpressing lines (S‐1, S‐2, S‐3) are shown. Scale bar = 1 cm. (H) Plant height measurement of different transgenic lines grown in soil for 1 month. (I) Lignin content in transgenic plants. (J) Endogenous jasmonic acid (JA) content in transgenic plants. (K) H_2_O_2_ levels in transgenic plants. (L) Leaf phenotypes of *SJP8*‐overexpressing and control (empty GFP vector) plants at 0 and 5 days post‐infiltration (dpi) with *Pseudomonas syringae* pv. *tomato* DC3000. Leaves collected from the same plants before infiltration (0 dpi) served as baseline controls for the 5 dpi samples. Three leaves per group (each from an independent line) represent three biological replicates. Scale bar = 1 cm. (M) Bacterial titres (CFU/cm^2^) in leaves at 5 dpi (corresponding to panel L). GFP indicates the control group. (N) Reverse transcription‐quantitative PCR analysis of *ZjLOX2* expression in leaves at 0 and 5 dpi. (O) Fold change of *ZjLOX2* expression at 5 dpi relative to 0 dpi (control), calculated from panel N. For panels (C), (H) and (N), statistical significance was determined by one‐way ANOVA; for panels (E, I, J, K, M, O), by Student's *t*‐test. Significance levels are indicated as follows: *p < 0.05, **p < 0.01, ***p < 0.001, ****p < 0.0001. Data are presented as mean ± SD of three technical replicates. *ZjActin* was used as an internal reference gene. All experiments were repeated three times independently with consistent results.

To investigate the biological function of SJP8 in jujube, we generated transgenic lines of Jingzao 39 overexpressing *SJP8* (Figure 2A,B). Relative to the empty‐vector (GFP) controls, these transgenic plants exhibited significantly reduced shoot length and markedly decreased cell length and width in stem cross‐sections (Figure 2C–E). After 28 days, both *SJP8*‐overexpressing and control plants developed well‐established root systems; although primary root length was shorter in the transgenic lines, total root number did not differ significantly (Figure S4a–d). One month after transfer to soil, control plants grew vigorously, whereas transgenic plants displayed chlorosis in apical leaves, significantly reduced plant height and decreased lignin content (Figure 2F–I). After 2 months, all *SJP8*‐overexpressing plants died, while control plants remained lush, vigorous and green. These results suggest that *SJP8* overexpression induces dwarfism and leaf chlorosis in transgenic jujube, ultimately leading to plant death.

To investigate whether SJP8 affects jujube immunity, we measured JA and H_2_O_2_ levels. Compared with controls, *SJP8*‐overexpressing plants showed significantly reduced JA content and elevated H_2_O_2_ levels (Figure 2J,K). To assess the functional consequences of JA suppression, we inoculated leaves of *SJP8*‐overexpressing Jingzao 39 plantlets with *Pseudomonas syringae* pv. *tomato* DC3000, a well‐established model for dissecting plant–pathogen interactions (Xin and He 2013; Wei and Collmer 2018; Jia et al. 2025). At 5 dpi, *SJP8*‐overexpressing leaves developed more severe chlorosis than controls, and bacterial titres were significantly higher in *SJP8*‐overexpressing lines (Figure 2L,M). RT‐qPCR analysis revealed that the JA‐related gene *ZjLOX2* (*Zj01G002900*) was significantly downregulated in *SJP8*‐overexpressing plants both before (0 dpi) and after (5 dpi) inoculation. Moreover, the fold change of *ZjLOX2* expression (5 dpi/0 dpi) was greater in *SJP8*‐overexpressing lines than that in GFP control (Figure 2N,O).

To explore whether the observed H_2_O_2_ elevation correlates with disease progression under natural conditions, we measured H_2_O_2_ levels in field‐grown jujube leaves at different stages of JWB disease (categorised as slight, moderate, or severe based on chlorosis severity). H_2_O_2_ content was significantly lower in mildly and moderately infected leaves than in healthy controls but became markedly elevated in severely infected leaves (Figure S5a,b). The pattern of H_2_O_2_ changes in the most severely chlorotic plants mirrored that observed in *SJP8*‐overexpressing plants. These results thus indicate that *SJP8* overexpression affects plant immune regulation.

### 2.3 *SJP8* Overexpression Induces Dwarfing in *Nicotiana tabacum* and *A. thaliana*

To investigate the function of SJP8, we generated T_1_ transgenic *N. tabacum* overexpressing *SJP8* (Figure S6a,b). Physiological assessment showed that the transgenic plants were significantly shorter than the controls (Figure S6c). We then measured lignin content in *SJP8*‐overexpressing plants and found a significant reduction compared with the control (Figure S6d). Although leaf number did not differ, leaves from the fourth layer upward were markedly smaller in *SJP8*‐overexpressing plants than in the control (Figure S6e–g). To determine whether *SJP8* overexpression affects plant immunity, we examined the expression of genes involved in JA biosynthesis, metabolism and signal transduction in leaves of transgenic plants by RT‐qPCR. These genes were significantly downregulated in *SJP8*‐overexpressing plants compared with the control (Figure S6h–j). Additionally, genes associated with H_2_O_2_ production, scavenging and signal transduction were significantly upregulated (Figure S6k–m).

In addition, we stably transformed SJP8 into *A. thaliana* . Dwarfism was evident in T_1_ transgenic plants (Figure S7a). Western blotting analysis confirmed SJP8 production in the T_3_ generation, and T_3_ plants had shorter roots than controls (Figure S7b–d). Three weeks after transplantation, the experimental group showed slower shoot and overall growth (Figure S7e). *SJP8*‐overexpressing plants were significantly shorter, produced more rosette leaves and exhibited delayed flowering and pod formation compared with GFP controls (Figure S7f–h). Stem cross‐sections revealed significantly reduced cell length and width in *SJP8*‐overexpressing plants compared with controls, and lignin content was also markedly decreased (Figure S7i–k). RT‐qPCR analysis of leaves showed that JA pathway genes were downregulated, whereas H_2_O_2_ pathway genes were upregulated (Figure S7l,m). Therefore, these results indicate that overexpression of *SJP8* induces dwarfism and modulates plant immunity in transgenic plants.

### 2.4 Transcriptome Analysis Reveals SJP8 Modulation of JA and H_2_O_2_ Pathways in Jujube and *Arabidopsis*

**FIGURE 3:**
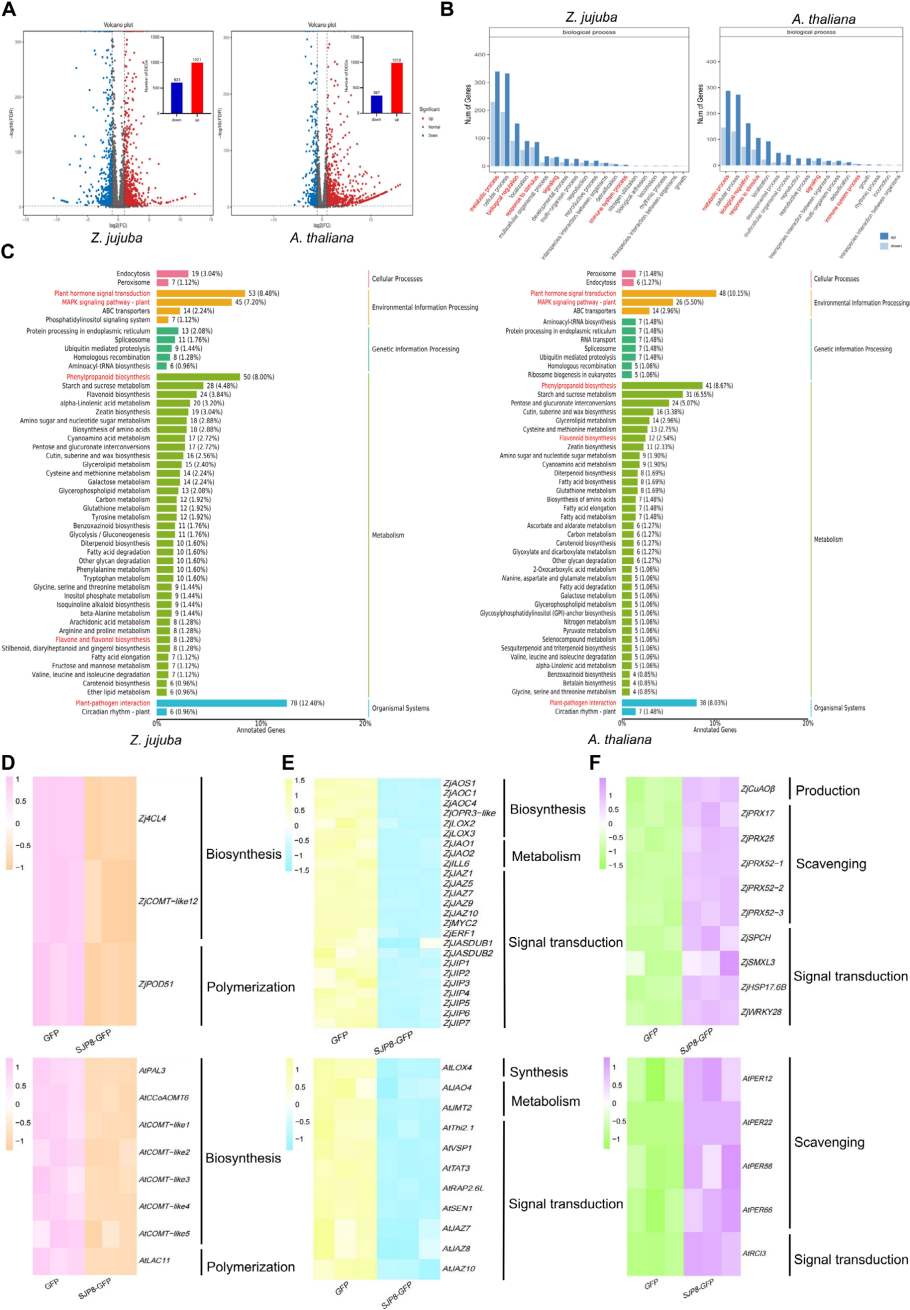
Transcriptome analysis of *SJP8*‐overexpressing *Ziziphus jujuba* ‘Jingzao 39’ and *Arabidopsis thaliana* reveals altered immunity‐associated genes and lignin synthesis. (A) Volcano plots of differentially expressed genes (DEGs) from RNA‐seq analysis of *Z. jujuba* (left) and *A. thaliana* (right). The bar graphs indicate the numbers of upregulated and downregulated DEGs for each species. (B) GO enrichment analysis (left: *Z. jujuba* ; right: *A. thaliana* ). (C) KEGG pathway enrichment analysis (left: *Z. jujuba* ; right: *A. thaliana* ). In panels (B) and (C), pathways related to plant immunity are highlighted in red. (D) Heatmaps of DEGs involved in lignin biosynthesis and polymerisation (upper: *Z. jujuba* ; lower: *A. thaliana* ). (E) Heatmaps of DEGs involved in jasmonic acid (JA) biosynthesis, metabolism and signal transduction (upper: *Z. jujuba* ; lower: *A. thaliana* ). (F) Heatmaps of DEGs involved in H_2_O_2_ production, scavenging and signal transduction (upper: *Z. jujuba* ; lower: *A. thaliana* ).

To investigate whether *SJP8* overexpression affects transcriptional regulation in transgenic plants, we performed transcriptome analysis on *SJP8*‐overexpressing Jingzao 39 and *A. thaliana* (Tables S2 and S3). To validate the transcriptome data, we examined the expression trends of selected genes (*ZjKTI5*, *ZjFra a 1.05*, *ZjPSK6* and *ZjGPT2* in Jingzao39; *AtDR4*, *AtEPR1*, *AtGASA4* and *AtCRA1* in *A. thaliana* ) by RT‐qPCR, which were consistent with the RNA‐seq results (Figure S8a,b). In total, 1652 differentially expressed genes (DEGs) (1021 upregulated and 631 downregulated) were identified in transgenic Jingzao 39 compared with the control group, and 1377 DEGs (1010 upregulated and 367 downregulated) were identified in transgenic *A. thaliana* compared with the control group (Figure 3A). Gene Ontology (GO) enrichment analysis of the biological process category showed that the DEGs in both species were mainly involved in metabolic process, biological regulation, response to stimulus, signalling and immune system processes, indicating their involvement in plant immune responses (Figure 3B, Tables S4 and S5). Kyoto Encyclopedia of Genes and Genomes (KEGG) analysis revealed that the DEGs were primarily enriched in pathways such as plant hormone signal transduction, MAPK signalling, phenylpropanoid biosynthesis, flavonoid biosynthesis and plant–pathogen interactions in both Jingzao 39 and *A. thaliana* (Figure 3C, Tables S6 and S7).

Given that *SJP8* overexpression reduced lignin content in both transgenic Jingzao 39 and *A. thaliana* , we found that the expression of DEGs involved in lignin biosynthesis and polymerisation was consistently downregulated in both species (Figure 3D, Tables S8 and S9). Regarding plant immunity, *SJP8* overexpression led to decreased JA levels and increased H_2_O_2_ levels in both transgenic Jingzao 39 and *A. thaliana* . Accordingly, DEGs involved in JA biosynthesis, metabolism and signal transduction were downregulated, whereas those associated with H_2_O_2_ production, scavenging and signal transduction were upregulated (note that no DEGs related to H_2_O_2_ production were identified in *A. thaliana* ) (Figure 3E,F, Tables [Link], [Link]). Collectively, these results suggest that *SJP8* overexpression may affect plant growth, development and immune regulation by interfering with lignin, JA and H_2_O_2_ pathways.

### 2.5 SJP8 Interacts With and Promotes Degradation of AtMYB61 via the 26S Proteasome Pathway

**FIGURE 4:**
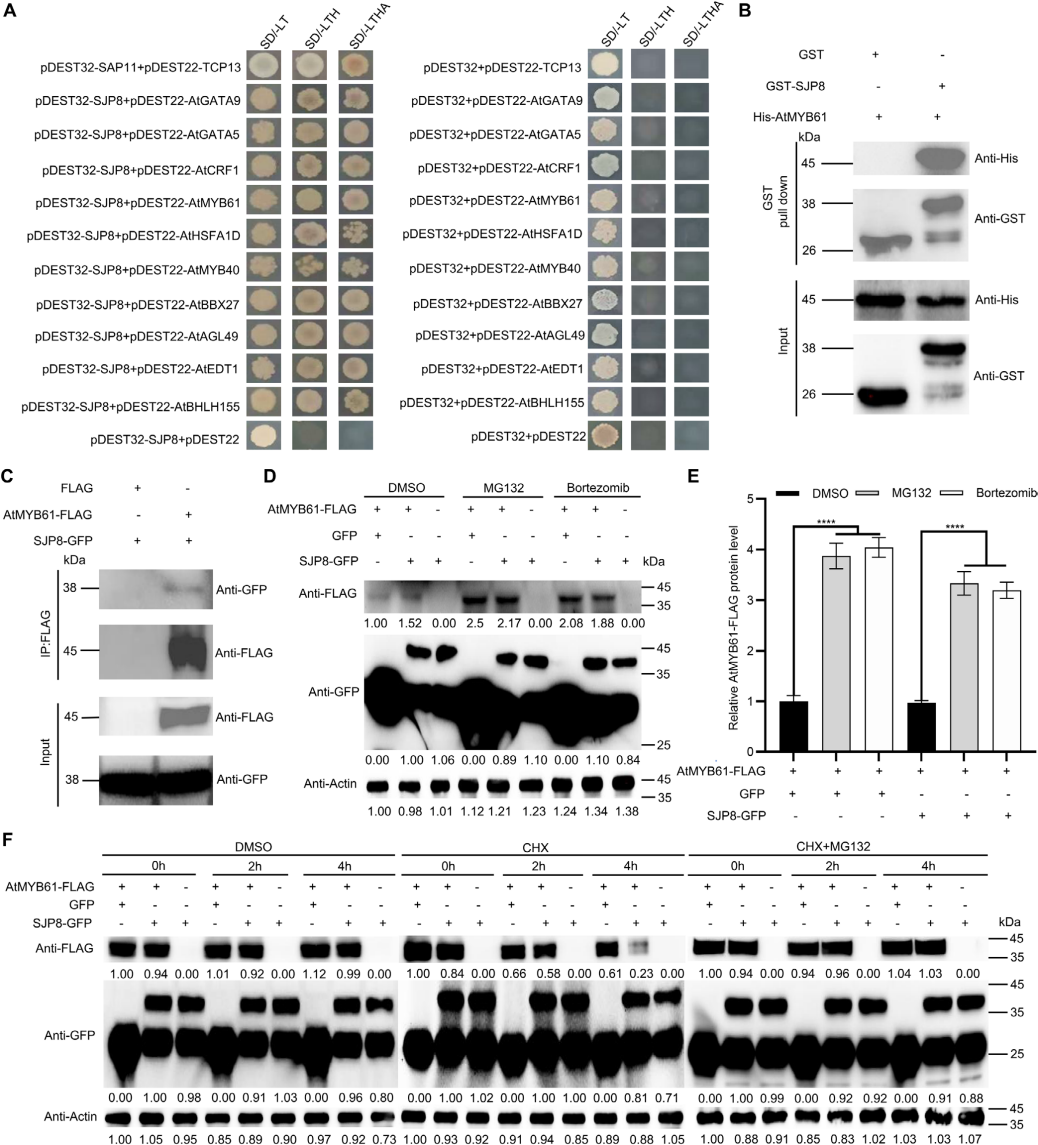
SJP8 interacts with and promotes the degradation of AtMYB61 via the 26S proteasome pathway. (A) Yeast two‐hybrid (Y2H) screening identified 10 *Arabidopsis thaliana* transcription factors that interact with SJP8. The pDEST32‐SAP11 + pDEST22‐TCP13 combination served as a positive control; empty pDEST22 and pDEST32 vectors were used as negative controls. SD/−LT, SD/−Trp−Leu; SD/−LTH, SD/−Trp−Leu−His with 10 mM 3‐AT; SD/−LTHA, SD/−Trp−Leu−His−Ade. (B) Glutathione S‐transferase (GST) pull‐down assay validating the interaction between SJP8 and AtMYB61. (C) Co‐immunoprecipitation (Co‐IP) assay confirming the SJP8–AtMYB61 interaction. In (B) and (C), ‘+’ and ‘–’ denote the presence and absence of the indicated components, respectively. Molecular weight markers (kDa) are shown on the left of each blot. (D) SJP8 destabilises AtMYB61 via the 26S proteasome pathway. Leaves co‐expressing *SJP8* and *AtMYB61* were treated with dimethyl sulphoxide (DMSO, control), MG132 or Bortezomib. In the immunoblot, ‘–’ and ‘+’ indicate the absence or presence of SJP8 and the inhibitors. Actin levels were used as a loading control. Band intensities were quantified and expressed relative to the DMSO‐treated control, which was set to 1.00. (E) Quantification of AtMYB61‐FLAG protein levels in (D). Band intensities were normalised to Actin. Data are presented as mean ± SD of three replicates and statistical significance was determined by one‐way ANOVA (****p < 0.0001). (F) Cycloheximide (CHX) chase assay. Total proteins were extracted from leaves treated with DMSO, CHX, or CHX + MG132 at the indicated time points and analysed by immunoblotting with anti‐FLAG, anti‐GFP and anti‐Actin antibodies. ‘–’ and ‘+’ indicate the absence or presence of the indicated treatments. Actin served as a loading control. Band intensities were quantified and expressed relative to the DMSO‐treated control (0 h), which was set to 1.00. Molecular weight markers (kDa) are shown on the right of each blot.

To investigate the pathogenic mechanism of SJP8, we performed yeast two‐hybrid (Y2H) screening using an *A. thaliana* cDNA library and identified 10 interacting transcription factors (Figure 4A, Table S14). Five of these interactors—AtbHLH155, AtHSF1D, AtMYB61, AtBBX27 and AtEDT1—were further validated by split‐luciferase complementation (split‐LUC) assay (Figure S9a,b). To determine whether the expression of these transcription factors is affected by SJP8, we performed RT‐qPCR on *SJP8*‐overexpressing transgenic *Arabidopsis*. The results showed that *AtbHLH155*, *AtHSF1D* and *AtBBX27* were upregulated, whereas *AtMYB61* and *AtEDT1* were downregulated (Figure S10). To identify the corresponding interacting partners in jujube, the natural host, we conducted gene family analysis and phylogenetic reconstruction for these five transcription factors (Figure S11). Using Y2H and split‐LUC assays, we identified the jujube homologues of AtbHLH155, AtHSF1D, AtMYB61, AtBBX27 and AtEDT1 as interactors of SJP8 (Figure S12, Table S15). Together, these results establish the biological relevance of the interactions between SJP8 and host transcription factors from the model plant *Arabidopsis* to the natural host jujube.

*AtMYB61*‐overexpressing *Arabidopsis* grow faster than wild‐type plants, with longer roots and a higher xylem‐to‐phloem ratio, whereas *atmyb61* loss‐of‐function mutants exhibit the opposite phenotype (Romano et al. 2012). In addition, *VvMYB15* and *VvMYB61* are co‐expressed with genes involved in phenylpropanoid biosynthesis and metabolic pathways, suggesting that these MYBs may regulate such pathways in grapevine in response to biotic stress (Zinati et al. 2024). Based on these findings, we selected AtMYB61 as a key interactor of interest to investigate the pathogenic mechanism of SJP8. We validated the interaction between AtMYB61 and SJP8 using glutathione S‐transferase (GST) pull‐down and co‐immunoprecipitation (Co‐IP) assays (Figure 4B,C). Given that SJP8 interacts with AtMYB61 and negatively regulates its transcription, we next investigated whether SJP8 also affects AtMYB61 protein stability. In vivo degradation assays in *N. benthamiana* leaves co‐expressing *SJP8* and *AtMYB61* showed that treatment with the 26S proteasome inhibitors MG132 or Bortezomib markedly increased AtMYB61 abundance relative to the dimethyl sulphoxide (DMSO) control (Figure 4D,E). A cycloheximide (CHX) chase assay further revealed that AtMYB61‐FLAG turned over more rapidly in the presence of SJP8 than in the empty vector (EV) control, with significantly less protein remaining at 4 h. Co‐treatment with MG132 largely prevented this SJP8‐induced degradation (Figure 4F, Figure S13). Collectively, these results demonstrate that SJP8 promotes the degradation of AtMYB61‐FLAG via the 26S proteasome pathway.

### 2.6 ZjMYB15 and ZjMYB86‐like Interact With Distinct Domains of SJP8

**FIGURE 5:**
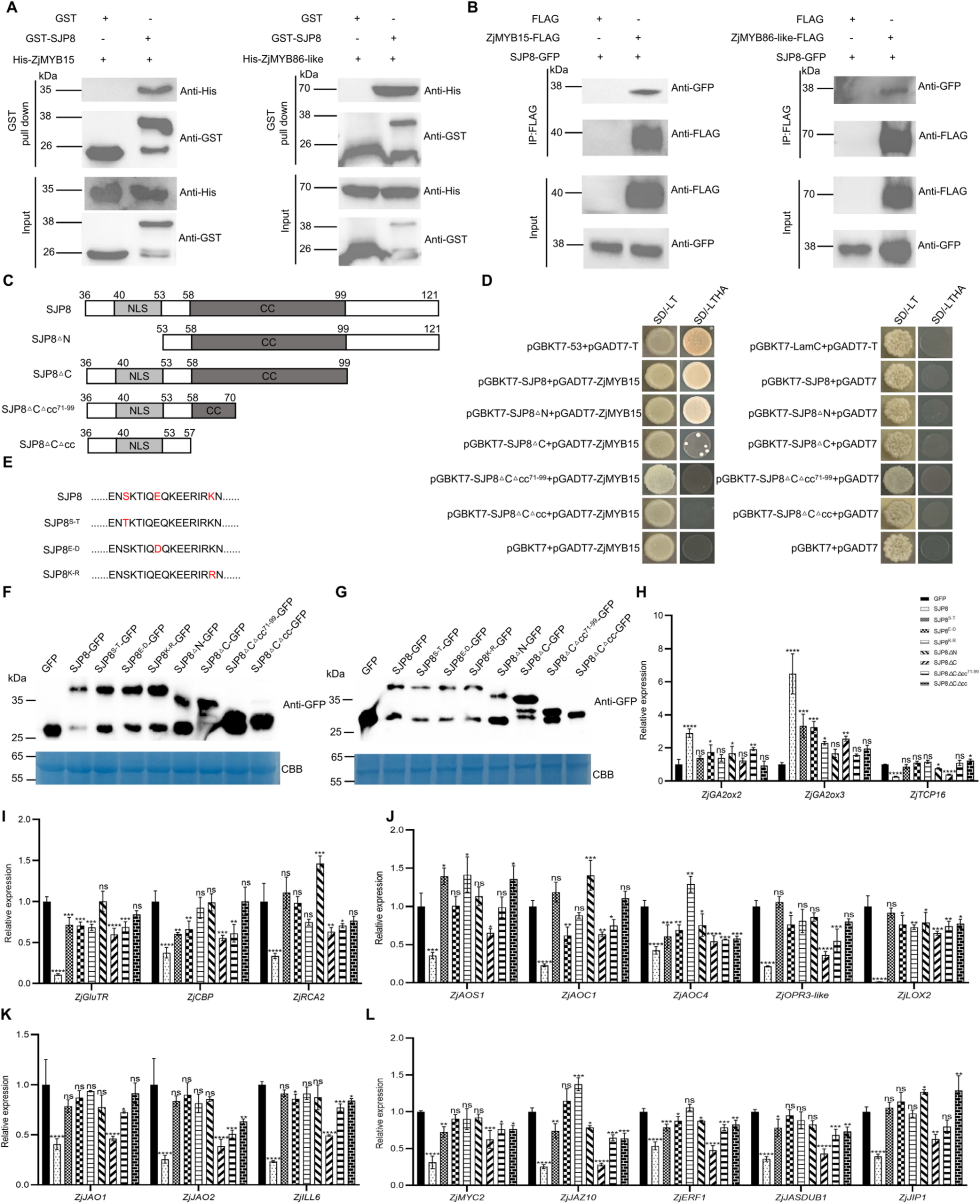
ZjMYB15 and ZjMYB86‐like interact with distinct domains of SJP8. (A) Glutathione S‐transferase (GST) pull‐down assay showing the interaction of SJP8 with ZjMYB15 and ZjMYB86‐like. ‘−’ and ‘+’ indicate absence and presence of the indicated components, respectively. (B) Co‐immunoprecipitation (Co‐IP) assay confirming the interaction of SJP8 with ZjMYB15 and ZjMYB86‐like. ‘−’ indicates absence and ‘+’ indicates presence of the indicated components. (C) Schematic representation of *SJP8* deletion mutants. Triangles indicate deleted regions. (D) Yeast two‐hybrid (Y2H) assay validating the interaction sites using the deletion mutant constructs shown in (C). pGBKT7‐53 + pGADT7‐T served as positive controls; pGBKT7‐LamC + pGADT7‐T, pGBKT7 and pGADT7 were used as negative controls. SD/−LT: SD/−Trp−Leu; SD/−LTHA: SD/−Trp−Leu−His‐−Ade. (E) Point mutations at the N‐terminus of SJP8. ‘SJP8S‐T’ indicates a single mutation of serine at the 38th amino acid position to threonine (AGT mutated to ACT). ‘SJP8E‐D’ indicates a single mutation of glutamic acid at the 46th amino acid position to aspartic acid (GAA mutated to GAC). ‘SJP8K‐R’ indicates a single mutation of lysine at the 51st amino acid position to arginine (AAA mutated to AGA). (F) Western blot detection of SJP8 and its deletion mutants in leaves of Jingzao 39 at 72 h post‐infiltration (hpi). (G) Western blot detection of SJP8 and its deletion mutants in stems of Jingzao 39 at 72 hpi. The large subunit of RuBisCO (visualised by Coomassie brilliant blue [CBB] staining) served as a loading control. Molecular weight markers (kDa) are indicated on the left. (H) Relative expression levels of dwarfing‐related genes (*ZjGA2ox2*, *ZjGA2ox3* and *ZjTCP16*) in stems of Jingzao 39 at 72 hpi, determined by reverse transcription‐quantitative PCR (RT‐qPCR). (I) Relative expression levels of chlorosis‐related genes (*ZjGluTR*, *ZjCBP* and *ZjRCA2*) in leaves of Jingzao 39 at 72 hpi, determined by RT‐qPCR. (J–L) Relative expression levels of jasmonic acid (JA) biosynthesis (J), metabolism (K) and signal transduction (L) genes in Jingzao 39 leaves at 72 hpi, determined by RT‐qPCR. For panels (H–L), statistical significance was assessed using one‐way ANOVA. *ZjActin* was used as an internal reference gene. Error bars represent the SD of three technical replicates. Significance levels are indicated as follows: ns, not significant *p* > 0.05, *p < 0.05, **p < 0.01, ***p < 0.001, ****p < 0.0001.

Gene family analysis, Y2H, and split‐LUC assays confirmed that SJP8 interacts with the jujube homologues of AtMYB61, ZjMYB15 and ZjMYB86‐like. The GST pull‐down and Co‐IP assays further validated the interaction between SJP8 and ZjMYB15 and ZjMYB86‐like (Figure 5A,B). To elucidate the interaction sites of SJP8 within ZjMYB15 and ZjMYB86‐like, we performed homology alignment of the protein sequences of SJP8 and SAP11_AY‐WB_ to identify the nuclear localisation sequence (NLS) and N‐terminal region of SJP8 (Figure S14a) (Sugio et al. 2014). Based on its structural domains, SJP8 was truncated into SJP8ΔN, SJP8ΔC and SJP8ΔCΔcc (Figure 5C). Importantly, Y2H and split‐LUC assays showed that only the SJP8 truncation lacking the coiled coil (CC) domain or containing a shorter CC domain (SJP8ΔCΔcc^71‐99^) failed to interact with ZjMYB15, indicating that the CC domain is necessary for the interaction between ZjMYB15 and SJP8 (Figure 5D, Figure S14b). Further Y2H and split‐LUC assays investigating the interaction between ZjMYB86‐like and the SJP8 deletion mutants revealed the strongest interaction with SJP8ΔCΔcc, whereas deletion of the N‐terminus resulted in no interaction (Figure S14c–d). To verify the specificity of the interaction between the N‐terminus of the SJP8 and ZjMYB86‐like, single base substitutions were applied in three different amino acid positions, with the 38th amino acid (serine) altered to produce threonine (AGT to ACT), the 46th amino acid (glutamate) altered to aspartic acid (GAA to GAC) and the 51st amino acid (lysine) to arginine (AAA to AGA) (Figure 5E). Y2H and split‐LUC assays demonstrated that ZjMYB86‐like did not interact with any of the three SJP8 mutants (Figure S14c–d). Collectively, these results indicate that ZjMYB15 specifically interacts with the CC domain of SJP8, whereas ZjMYB86‐like specifically interacts with the N‐terminus of SJP8.

To assess the functional relevance of the identified domains, we generated a series of SJP8 deletion and point mutants (*35S::SJP8* ^*S‐T*^, *35S::SJP8* ^*E‐D*^, *35S::SJP8* ^*K‐R*^, *35S::SJP8ΔN*, *35S::SJP8ΔC*, *35S::SJP8ΔCΔcc* ^*71–99*^ and *35S::SJP8ΔCΔcc*) and transiently transformed wild‐type Jingzao 39 tissue‐cultured seedlings. Western blotting confirmed expression of all constructs in leaves and stems at 3 days post‐transformation (Figure 5F,G). RT‐qPCR analysis showed that seedlings expressing *SJP8* or *SJP8ΔC* exhibited significant upregulation of dwarfing‐related genes (*ZjGA2ox2*, *ZjGA3ox*) and downregulation of *ZjTCP16*, as well as downregulation of chlorosis‐related genes (*ZjGluTR*, *ZjCBP*, *ZjRCA2*). In contrast, seedlings expressing the other mutants showed no or only weak changes in these genes (Figure 5H,I). Regarding immune regulation, seedlings expressing *SJP8* or *SJP8ΔC* displayed significant downregulation of JA biosynthesis, metabolism and signal transduction genes, and upregulation of H_2_O_2_‐related genes (production, scavenging and signalling), whereas the other mutants had little effect (Figure 5J–L, Figure S15a). Similar results were obtained upon transient transformation of *N. benthamiana* (Figure S15b–h). Collectively, these findings demonstrate that the N‐terminal and CC domains of SJP8 are required for SJP8‐induced changes in the expression of marker genes associated with dwarfism, chlorosis and immunity.

### 2.7 SJP8 Promotes the Degradation of ZjMYB15 and ZjMYB86‐like via the 26S Proteasome Pathway

Considering that SJP8 interacts with ZjMYB15 and ZjMYB86‐like, we examined their expression levels by RT‐qPCR. Both *ZjMYB15* and *ZjMYB86‐like* were downregulated in *SJP8*‐overexpressing jujube (Figure S16a). Consistently, their expression was also reduced in both leaves and stems of jujube plants infected with JWB disease (Figure S16b,c). These results suggest that ZjMYB15 and ZjMYB86‐like may play critical roles during JWB infection.

**FIGURE 6:**
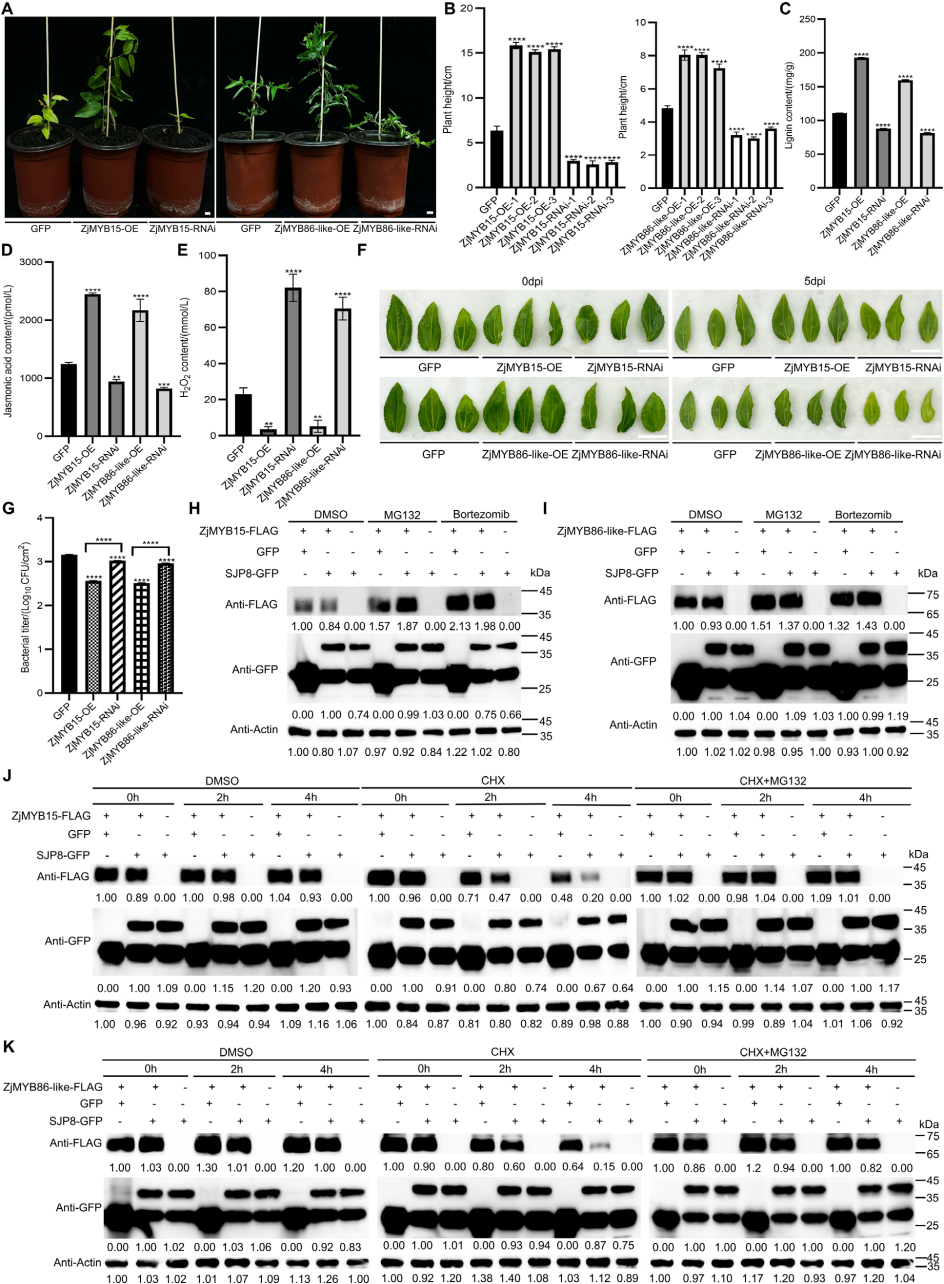
ZjMYB15 and ZjMYB86‐like promote plant growth and defence, and are targeted for degradation by SJP8 via the 26S proteasome. (A) Plant height phenotypes of overexpression (OE) and RNAi lines grown in soil for 2 months. Scale bar = 1 cm. (B) Quantification of plant height for the lines shown in (A). (C) Lignin content in stems of the transgenic lines. (D) Jasmonic acid (JA) content in the transgenic lines. (E) H_2_O_2_ levels in the transgenic lines. (F) Leaf phenotypes of overexpression (OE) and silencing (RNAi) lines at 0 and 5 days post‐inoculation (dpi) with *Pseudomonas syringae* pv. *tomato* DC3000. Leaves collected before infiltration (0 dpi) from the same plants served as baseline controls. Three independent transgenic lines per construct were used as biological replicates. Scale bar = 1 cm. (G) Bacterial titres (CFU/cm^2^) in leaves at 5 dpi, corresponding to (F). GFP‐expressing plants served as controls. (H, I) Immunoblot analysis of ZjMYB15‐FLAG (H) and ZjMYB86‐like‐FLAG (I) levels in leaves co‐expressing *SJP8* with the respective targets, treated with dimethylsulphoxide (DMSO, control), 50 μM MG132, or 50 μM Bortezomib. (J, K) Cycloheximide (CHX) chase assay for ZjMYB15 (J) and ZjMYB86‐like (K). Total proteins were extracted at the indicated time points from leaves treated with DMSO, CHX, or CHX + MG132 and analysed by immunoblotting using anti‐FLAG, anti‐GFP and anti‐Actin antibodies. In panels (H–K), ‘–’ and ‘+’ indicate the absence or presence of the specified components or treatments. Actin levels were used as a loading control. Band intensities were quantified and expressed relative to the DMSO‐treated control, which was set to 1.00. Molecular weight markers (kDa) are shown on the right of each blot. For panels (B–G), data are presented as mean ± SD (*n* = 3). Statistical significance was determined by one‐way ANOVA **p < 0.01, ***p < 0.001, ****p < 0.0001.

To explore the biological functions of ZjMYB15 and ZjMYB86‐like, subcellular localisation assays showed that both proteins were localised in the nucleus (Figure S16d). Genetic transformation of Jingzao 39 with *ZjMYB15* and *ZjMYB86‐like* generated three overexpression and three RNAi positive transgenic lines for each gene, and RT‐qPCR confirmed altered mRNA expression levels in these backgrounds (Figure S16e–f). After 3 weeks of in vitro culture, overexpression lines exhibited enhanced shoot elongation, whereas RNAi lines showed reduced elongation compared with the control (Figure S17a,b). Furthermore, when soil‐grown plants were examined after 2 months, both OE lines displayed a pronounced increase in plant height, whereas the RNAi lines were significantly shorter than the control, indicating that these transcription factors positively regulate plant growth (Figure 6A,B). In addition, lignin content was significantly increased in OE lines and decreased in RNAi lines compared with the control (Figure 6C). Taken together, overexpression of *ZjMYB15* or *ZjMYB86‐like* promotes plant height and lignin accumulation, whereas RNAi lines show the opposite phenotype.

To further investigate whether ZjMYB15 and ZjMYB86‐like are involved in plant immunity, we performed RT‐qPCR analysis. Compared with controls, plants overexpressing *ZjMYB15* and *ZjMYB86‐like* showed upregulation of JA‐related genes *ZjLOX2* and downregulation of H_2_O_2_‐related genes *ZjCuAOβ* (*Zj07G013500*). Conversely, RNAi lines exhibited opposite expression patterns (Figure S18a,b). Accordingly, overexpression lines accumulated higher JA and lower H_2_O_2_ levels, whereas RNAi lines showed reduced JA and elevated H_2_O_2_ levels (Figure 6D,E). Collectively, these results demonstrate that ZjMYB15 and ZjMYB86‐like positively regulate JA content and negatively regulate H_2_O_2_ accumulation. To assess the functional consequences of this JA promotion, we inoculated overexpression and RNAi lines of *ZjMYB15* and *ZjMYB86‐like* with *P. syringae* pv. *tomato* DC3000. At 5 days post‐inoculation, leaves of the overexpression lines remained visibly greener than controls, whereas RNAi lines exhibited more severe yellowing (Figure 6F). Bacterial titres were quantified, and both overexpression lines supported significantly lower bacterial growth compared with controls, while RNAi lines showed higher bacterial loads than overexpression lines (Figure 6G). RT‐qPCR analysis revealed that the JA‐related gene *ZjLOX2* was significantly upregulated in overexpression lines but downregulated or unchanged in RNAi lines upon *P. syringae* pv. *tomato* DC3000 infection, both before (0 dpi) and after (5 dpi) inoculation (Figure S19a–c). Moreover, the fold change of *ZjLOX2* expression (5 dpi/0 dpi) was greater in both *ZjMYBs*‐overexpressing and *ZjMYBs*‐RNAi lines than in empty vector controls (Figure S19b,d). Collectively, these results indicate that ZjMYB15 and ZjMYB86‐like promote resistance to bacterial infection by enhancing JA‐mediated immunity.

Given that ZjMYB15 and ZjMYB86‐like act as positive immune regulators, we tested whether SJP8 affects their protein stability. Co‐expression of *GFP‐SJP8* with FLAG‐tagged *ZjMYB15* or *ZjMYB86‐like* in *N. benthamiana* leaves, followed by MG132 or Bortezomib treatment, led to a clear accumulation of both proteins relative to the DMSO control, indicating basal 26S proteasome‐dependent turnover (Figure 6H,I, Figure S20a,b). CHX chase assays further revealed that both proteins were degraded considerably faster in the presence of SJP8 than in the EV control, with markedly less protein remaining at 4 h. This accelerated degradation was largely blocked by co‐treatment with MG132 (Figure 6J,K, Figure S21a,b). Collectively, these results demonstrate that SJP8 promotes the 26S proteasome‐dependent degradation of ZjMYB15 and ZjMYB86‐like.

### 2.8 ZjMYB15 and ZjMYB86‐like Positively Regulate the Transcription of *ZjJAIPHX1* and *ZjPOD43*

**FIGURE 7:**
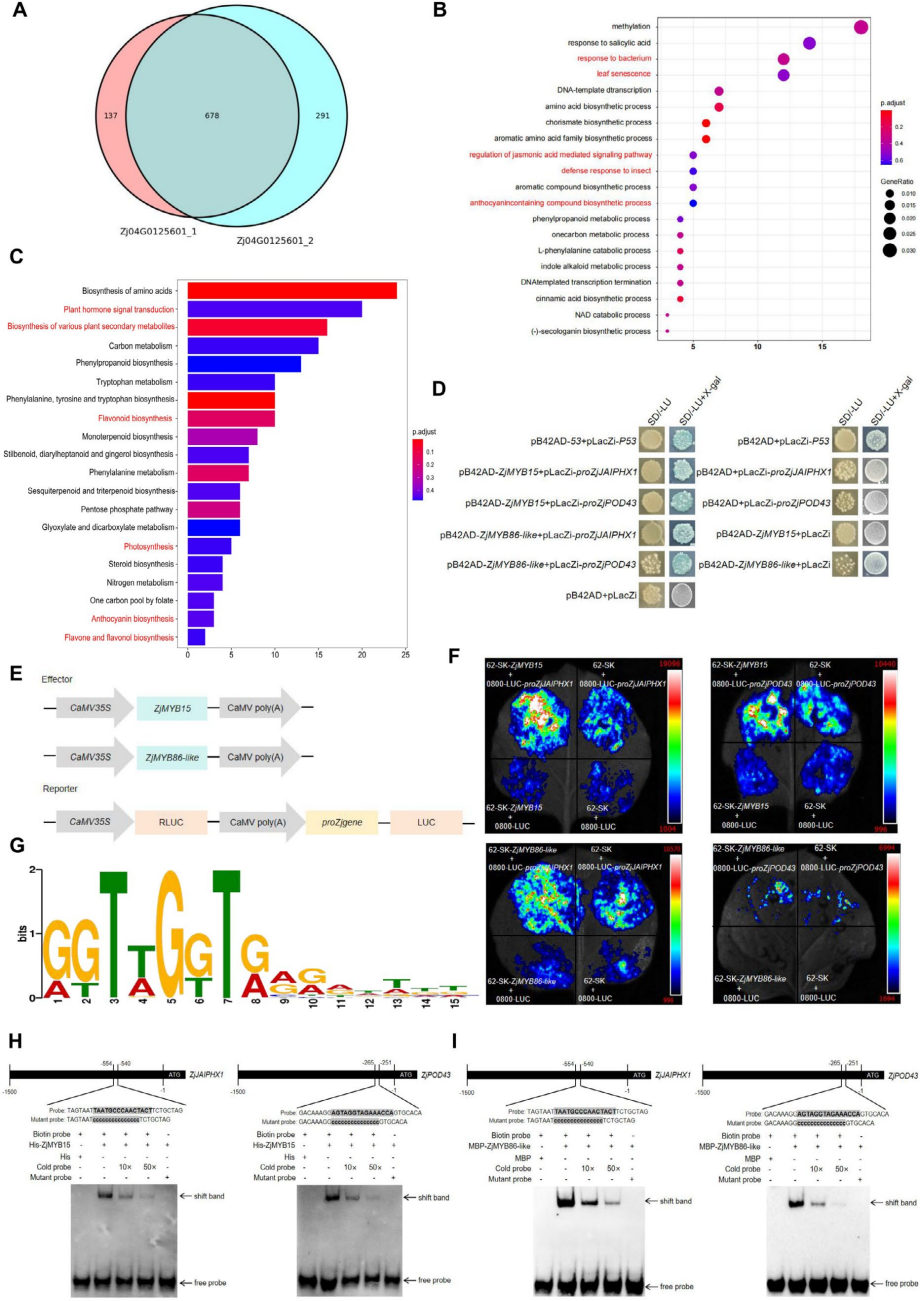
Identification of ZjMYB15 and ZjMYB86‐like target genes by DNA affinity purification (DAP)‐seq. (A) Venn diagram showing the overlap of peaks between two technical replicates of DAP‐seq. (B) Top 20 enriched GO terms (biological process category) among genes bound by ZjMYB15. (C) Top 20 enriched KEGG pathways among genes bound by ZjMYB15. (D) Yeast one‐hybrid assays validating the binding of ZjMYB15 and ZjMYB86‐like to the promoters of *ZjJAIPHX1* and *ZjPOD43*. pB42AD‐*53* + pLacZi‐*P53* served as a positive control; pB42AD + pLacZi‐*P53* and empty vectors were used as negative controls. SD/−LU: SD/−Leu − Ura; SD/−LU + X‐gal: SD/−Leu − Ura supplemented with X‐gal. (E) Schematic diagram of the dual‐luciferase reporter vector construction. (F) Dual‐luciferase reporter gene assays confirming the activation of *ZjJAIPHX1* and *ZjPOD43* promoters by ZjMYB15 and ZjMYB86‐like. pGreenII 62‐SK + pGreenII 0800 were used as negative controls. (G) Enriched DNA binding motif of ZjMYB15 within the promoters of *ZjPOD43* and *ZjJAIPHX1* identified by MEME‐ChIP. (H, I) Electrophoretic mobility shift assay (EMSA) showing that ZjMYB15 (H) and ZjMYB86‐like (I) bind to the TAATGCCCAACTACT motif of the *ZjJAIPHX1* promoter and the AGTAGGTAGAAACCA motif of the *ZjPOD43* promoter. Biotin‐labelled probes (biotin) and unlabelled competitive probes (cold, at 10‐ and 50‐fold excess) were used. His‐tagged ZjMYB15 and MBP‐tagged ZjMYB86‐like were purified; corresponding empty vectors served as negative controls. ‘−’ indicates absence and ‘+’ indicates presence of the indicated components.

To elucidate how ZjMYB15 and ZjMYB86‐like promote JA content and suppress H_2_O_2_ accumulation, and given that SJP8 exhibits a stronger interaction with ZjMYB15, we performed DNA affinity purification sequencing (DAP‐seq) to identify the direct downstream targets of ZjMYB15. Comparative analysis of two replicate DAP‐seq experiments identified 1106 promoters, of which 678 were co‐bound by ZjMYB15 (Figure 7A, Table S16). GO enrichment analysis of biological process revealed terms such as response to bacterium, leaf senescence, regulation of jasmonic acid‐mediated signalling pathway, defence response to insect and anthocyanin‐containing compound biosynthetic process (Figure 7B). KEGG enrichment analysis showed pathways including plant hormone signal transduction, biosynthesis of various plant secondary metabolites, flavonoid biosynthesis, photosynthesis, anthocyanin biosynthesis and flavone and flavonol biosynthesis (Figure 7C). Overall, these results indicate that ZjMYB15 is involved in regulating plant growth, development and immunity.

Based on functional annotation of the target genes pulled down by DAP‐seq, we identified two genes related to JA and H_2_O_2_ pathways: *ZjJAIPHX1* (*Zj10G000470*, *jasmonate‐induced protein homologue isoform X1*) and *ZjPOD43* (*Zj09G014410*, *peroxidase 43*) (Tables S17 and S18). To determine their subcellular localisation, we performed subcellular localisation and plasmolysis assays. ZjPOD43 was localised in the cytoplasm, whereas ZjJAIPHX1 was localised in both the nucleus and cytoplasm (Figure S22a,b). Yeast one‐hybrid (Y1H) assay showed that ZjMYB15 activated the expression of both promoters, as evidenced by the growth and blue colouration of co‐transformed yeast strains on SD/−Leu−Ura plates containing X‐gal, confirming binding to *proZjJAIPHX1* and *proZjPOD43* (Figure 7D). Dual‐luciferase reporter assay performed after co‐injection of ZjMYB15 and the promoters into *N. benthamiana* leaves revealed that ZjMYB15 positively regulated the transcription of *ZjJAIPHX1* and *ZjPOD43* (Figure 7E,F, Figure S23a). Given the homology and close phylogenetic relationship between ZjMYB86‐like and ZjMYB15, we further confirmed by Y1H assay that ZjMYB86‐like also bound to *proZjJAIPHX1* and *proZjPOD43* (Figure 7D). Consistently, dual‐luciferase reporter assay demonstrated that ZjMYB86‐like promotes the transcription of both genes (Figure 7E,F, Figure S23b). Moreover, RT‐qPCR analysis showed that *ZjJAIPHX1* and *ZjPOD43* were upregulated in plants overexpressing *ZjMYB15* or *ZjMYB86‐like* and downregulated in RNAi lines (Figure S23c,d). Thus, ZjMYB15 and ZjMYB86‐like act as positive regulators of *ZjJAIPHX1* and *ZjPOD43*. Consistent with this regulation, RT‐qPCR analysis revealed that *ZjJAIPHX1* and *ZjPOD43* were downregulated in leaves and stems of JWB‐infected jujube (Figure S24a,b). Their homologues were also downregulated in *SJP8*‐overexpressing plants of four species (Figure S24c,d). Clustering analysis showed that *JAIPHX1* homologues clustered with JA‐related marker genes, indicating that SJP8 negatively regulates *JAIPHX1* and that *JAIPHX1* is involved in the JA pathway (Figure S24e, Table S19). In contrast, *POD43* homologues were downregulated despite upregulation of H_2_O_2_‐related marker genes (Figure S24f, Table S20), suggesting that SJP8 specifically suppresses *POD43* expression, which may impair H_2_O_2_ scavenging and contribute to H_2_O_2_ accumulation.

To further characterise the binding sites, DAP‐seq data revealed that the bound motif contains a palindromic 15‐bp conserved sequence (5′‐GGTWGGTRRRRWWDD‐3′) (Figure 7G). Electrophoretic mobility shift assay (EMSA) confirmed that ZjMYB15 and ZjMYB86‐like bind to the promoter regions of *ZjJAIPHX1* and *ZjPOD43*, with the binding site for *proZjJAIPHX1* being TAATGCCCAACTACT and for *proZjPOD43* being AGTAGGTAGAAACCA (Figure 7H,I). To assess conservation, we compared these two motifs with seven known AtMYB61 binding motifs in *A. thaliana* and found 27.16% sequence identity, with enrichment of AC sequences (Figure S25) (Prouse and Campbell 2013). This suggests that these binding sites are evolutionarily conserved.

Together, these results indicate that both ZjMYB15 and ZjMYB86‐like directly bind to the AGTAGGTAGAAACCA motif in *proZjPOD43* and the TAATGCCCAACTACT motif in *proZjJAIPHX1*, activating their expression. This in turn increases JA levels but decreases H_2_O_2_ accumulation, thereby affecting plant growth and development (dwarfism and chlorosis) as well as plant immunity.

## 3 Discussion

Effector proteins, which are crucial for pathogen virulence, are potential targets for controlling diseases associated with phytoplasmas (Tomkins et al. 2018; Galetto et al. 2021). For example, overexpression of *SJP1*_JWB_ and *SJP2*_JWB_ in both *N. benthamiana* and jujube promotes lateral bud outgrowth and induces witches' broom‐like symptoms (Zhou et al. 2021). In this study, overexpression of *SJP8* in Jingzao 39, *N. tabacum* and *A. thaliana* consistently induced dwarfism, indicating that SJP8 is a conserved effector affecting plant growth and immunity (Figures 2 and 3; Figure S6). Notably, leaf chlorosis occurred only in *SJP8*‐overexpressing jujube, the natural host, suggesting that this woody plant is more sensitive to SJP8. Similarly, overexpression of *SWP12*_*WBD*_ in the wheat cultivar Xiaoyan 6 (XY6) resulted in severe chlorosis compared to the control, indicating its role as a key phytopathogenic effector (Bai et al. 2022). Species‐specific differences likely reflect host‐adaptive strategies of ‘*Ca*. P. ziziphi’. Chlorosis and dwarfism are typical phytoplasma symptoms, and their differential manifestation indicates that SJP8 has evolved to optimally perturb jujube‐specific regulatory networks, possibly due to differences in host target abundance, functional divergence, or distinct hormonal microenvironments. Moreover, the expression levels of vector‐borne pathogen effectors differ between host and vector and are influenced by host genotype (Huang et al. 2020). Consequently, effector expression profiles vary among host genotypes, which correlates with differential levels of tolerance and resistance to phytoplasmas (Bertazzon et al. 2019; Shi et al. 2019). Therefore, these findings establish SJP8 as a key pathogenicity effector of ‘*Ca*. P. ziziphi’, corroborating and extending the work of Wan et al. (2025), who reported that SJP8 suppresses plant immunity and enhances phytoplasma proliferation in both *N. benthamiana* and *A. thaliana* (Wan et al. 2025).

Overexpression of *SJP8* affects not only plant growth and development but also plant immunity. RT‐qPCR analysis of leaves from *SJP8*‐overexpressing *N. benthamiana*, *N. tabacum* and *A. thaliana* consistently showed downregulation of JA‐related genes and upregulation of H_2_O_2_‐related genes. Transcriptome data from *SJP8*‐overexpressing *A. thaliana* and jujube confirmed the JA results. However, for the H_2_O_2_ pathway, only scavenging‐ and signalling‐related genes were differentially expressed in *A. thaliana*, possibly due to species differences and the use of stem tissues in both species. Furthermore, JA content was reduced and H_2_O_2_ levels were elevated in *SJP8*‐overexpressing plants of both species. Thus, SJP8 acts as a negative regulator of JA and a positive regulator of H_2_O_2_. Notably, the enriched KEGG and GO modules are consistent with those previously reported in the transcriptome of JWB‐infected jujube leaves and JWB790‐transgenic *Arabidopsis* inflorescence stems (Wang, Ye, et al. 2018; Wan et al. 2025). These consistent enrichment patterns further support the role of SJP8 in modulating plant immunity.

JA suppresses various aspects of seedling growth, including primary root elongation, leaf expansion and hypocotyl extension (Wasternack and Hause 2013; Kim et al. 2015; Huang et al. 2017). The dwarfism observed in *SJP8*‐overexpressing plants, as well as the shorter roots of *SJP8*‐overexpressing *Arabidopsis*, may also be linked to JA. Moreover, *SJP8*‐overexpressing leaves inoculated with *P. syringae* pv. *tomato* DC3000 showed more severe chlorosis than controls, accompanied by downregulation of the JA‐related gene *ZjLOX2*. Therefore, we speculate that JA is a key hormone in plant defence against pathogens and that SJP8 may negatively regulate JA content. However, JA levels can also change dynamically during infection. For example, JA content in both genotypes (PZ and T13) increased during the first two growth stages (S1 and S2) after infection, reaching 110 mg/g at S2 and then decreased at S3 (Wang et al. 2022). However, whether SJP8 modulates plant growth and immunity through hormones other than JA is a key future direction for research on JWB pathogenesis.

H_2_O_2_ is generated via superoxide dismutase‐catalysed dismutation of superoxide, with superoxide originating from electron transport in chloroplasts and mitochondria, plasma membrane NADPH oxidases, peroxisomal oxidases, type III peroxidases and other apoplastic oxidases (Smirnoff and Arnaud 2019). ROS production was upregulated in phytoplasma‐infected jujube leaves, and higher activities of GST and peroxidase were detected in resistant cultivars, indicating that the antioxidant defence system may be involved in phytoplasma attack (Xue et al. 2020). Excessive ROS production can damage plant cells, whereas low ROS levels can activate phytoalexins and pathogenesis‐related genes to suppress pathogen development (Gao et al. 2018). In the present study, H_2_O_2_ levels were significantly upregulated in transgenic plants overexpressing *SJP8*. Considering that H_2_O_2_ is one of the signals for photosynthetic status and for stomatal movements, excess H_2_O_2_ triggers chloroplast and peroxisome autophagy and programmed cell death (Smirnoff and Arnaud 2019). SRP1_RDLP_ from rice orange leaf phytoplasma disrupts chloroplast glutamine synthetase stability, resulting in chlorosis (Zhang et al. 2024). Therefore, our findings indicate that overexpression of *SJP8* may lead to excessive H_2_O_2_ production, impairing the plant immune system, disrupting photosynthesis, inhibiting growth, inducing leaf chlorosis and ultimately causing plant death.

The signal peptide of SJP8 is functional, and SJP8 lacking the signal peptide localises to the nucleus and cytoplasm, consistent with previous reports (Wang, Yang, et al. 2018; Wan et al. 2025). Truncation analysis revealed that SJP8 interacts with ZjMYB15 via its CC domain, whereas the N‐terminal domain serves as the interaction site for ZjMYB86‐like, a domain‐specific binding pattern also observed in other effectors (Sugio et al. 2014). Single‐base mutations in the N‐terminal domain confirmed binding specificity, analogous to the key residues P85 and D33 of the phytoplasma effector SWP12_WBD_, which are required for target interaction and immune suppression (Bai et al. 2023). Mutation of key N‐terminal residues (S38, E46, K51) or disruption of the CC domain compromised the ability of SJP8 to induce changes in the expression of JA‐ and H_2_O_2_‐related genes, as well as genes associated with dwarfism and chlorosis, in both *N. benthamiana* and jujube Jingzao 39, indicating that these motifs are required for SJP8‐mediated transcriptional reprogramming linked to symptom development. Whether these mutants can attenuate or eliminate the developmental phenotypes of dwarfism and chlorosis awaits long‐term stable transformation in jujube, which remains an important direction for future investigation. These key residues may therefore represent candidate sites for future efforts to engineer disease‐resistant jujube varieties, and their identification advances our understanding of the molecular virulence mechanism of SJP8.

Lignin is a critical cell wall constituent that constitutes a physical barrier against pathogens; its downregulation impairs host defence and facilitates phytoplasma infection (Lee et al. 2019; Onohata and Gomi 2020; Kashyap et al. 2021). Many effectors suppress host growth and immunity by targeting host proteins (Anderson et al. 2015; Lan et al. 2019; Liu et al. 2024). Heterologous expression of *AtMYB61* in rice increases lignin content (Koshiba et al. 2017). Moreover, in rice, OsMYB110 negatively regulates plant height (Wang et al. 2024). In jujube, ZjMYB15 and ZjMYB86‐like positively regulate plant height and lignin content, whereas *SJP8* overexpression causes opposite effects, indicating that SJP8 degrades these positive regulators to reduce lignin and stunt growth. The ubiquitin‐proteasome system plays a key role in plant growth and immune modulation (Moon et al. 2004; Ding et al. 2022; Wang and Zeng 2022). Phylogenetic analysis linked SJP8 (JWB790) to a ubiquitin ligase, M19_00185 (Wan et al. 2025), and in vivo degradation assays confirmed that SJP8 mediates degradation of ZjMYB15 and ZjMYB86‐like via the 26S proteasome, providing mechanistic insight into how SJP8 interferes with plant growth and immunity.

Overexpression of *ZjMYB15* and *ZjMYB86‐like* in jujube upregulated JA‐related genes and increased JA content, whereas RNAi lines showed the opposite effects (Figure 7E,F). Consistently, in leaves transiently inoculated with *P. syringae* pv. *tomato* DC3000, overexpression lines exhibited greener leaves and elevated *ZjLOX2* expression, further confirming that these MYB transcription factors activate JA. Given that higher JA content enhances resistance to JWB phytoplasma (Wang et al. 2022), our results suggest a JA‐mediated defence mechanism. Additionally, overexpression of *ZjMYB15* and *ZjMYB86‐like* downregulated H_2_O_2_‐related genes and reduced H_2_O_2_ content. Supporting a broader role for MYB proteins, TaMYB86B is induced by multiple stresses and affects salt tolerance by maintaining osmotic balance and reducing ROS levels (Song et al. 2020), while MdMYB54 enhances cell wall defence via interaction with MdERF114 (Liu, Chen, et al. 2025). Collectively, these findings indicate that overexpression of *ZjMYB15* and *ZjMYB86‐like* modulates JA‐ and H_2_O_2_‐related gene expression, thereby regulating immune responses.

Therefore, phytoplasmas employ a diverse arsenal of effector proteins to hijack host processes. Known effectors target transcription factor families such as ARF, TCP, MADS‐box, SPL, GATA and WRKY to interfere with plant growth, development and immune regulation (Minato et al. 2014; Sugio et al. 2014; Huang et al. 2021; Bai et al. 2022; Orlovskis et al. 2025; Zhang, Li, Qiu, et al. 2025). Our finding that SJP8 targets MYB transcription factors for degradation adds a new layer to this model, with SJP8 acting on MYB proteins to induce dwarfism and chlorosis. This highlights a common theme of transcription factor hijacking while revealing remarkable versatility in specific host targets, enabling phytoplasmas to disrupt diverse physiological processes. An intriguing question is whether natural variation in ZjMYB15 and ZjMYB86‐like contributes to differential susceptibility among jujube cultivars. It is plausible that natural alleles encoding proteins with alterations in the SJP8‐interaction domain could confer resistance by evading effector binding and degradation. Future work should sequence these loci across resistant and susceptible varieties to identify such alleles, which would be invaluable for breeding durable resistance to JWB disease.

Plant cells have evolved efficient antioxidant systems, including peroxidases (PODs), to scavenge ROS (Smirnoff and Arnaud 2019). For instance, the ZjMYB44‐ZjPOD51 module enhances jujube defence against phytoplasma by upregulating *ZjPOD51* (Zhang, Li, Wei, et al. 2025). Based on the results of DAP‐seq experiments, we verified the downstream target genes of ZjMYB15 and ZjMYB86‐like through Y1H assay, dual‐luciferase reporter assay and EMSA. Moreover, R2R3‐MYB proteins are known to bind AC elements and activate transcription from these motifs in planta (Prouse and Campbell 2012, 2013; Guo et al. 2017; Liu, Li, et al. 2025). In this study, ZjMYB15 and ZjMYB86‐like directly bound to the AGTAGGTAGAAACCA motif of the promoter of *ZjPO43* and bound to the TAATGCCCAACTACT motif of the promoter of *ZjJAIPHX1* and activated their expression. All motifs exhibited AC‐rich regions, indicating evolutionary conservation of the ZjMYB15 and ZjMYB86‐like binding site. Furthermore, ZjMYB86‐like, a homologue of both ZjMYB15 and AtMYB61, was found to bind an identical motif, further supporting the functional conservation of MYB family protein binding motifs. In transgenic jujube overexpressing *ZjMYB15* or *ZjMYB86‐like*, *ZjJAIPHX1* and *ZjPOD43* were upregulated, coinciding with increased JA and decreased H_2_O_2_ levels. Conversely, both genes were downregulated in JWB‐infected jujube and in *SJP8*‐overexpressing plants across multiple species. Clustering analysis showed that SJP8 negatively regulates *JAIPHX1* and that *JAIPHX1* is involved in the JA pathway, and SJP8 specifically suppresses *POD43* expression, which may impair H_2_O_2_ scavenging and contribute to H_2_O_2_ accumulation.

**FIGURE 8:**
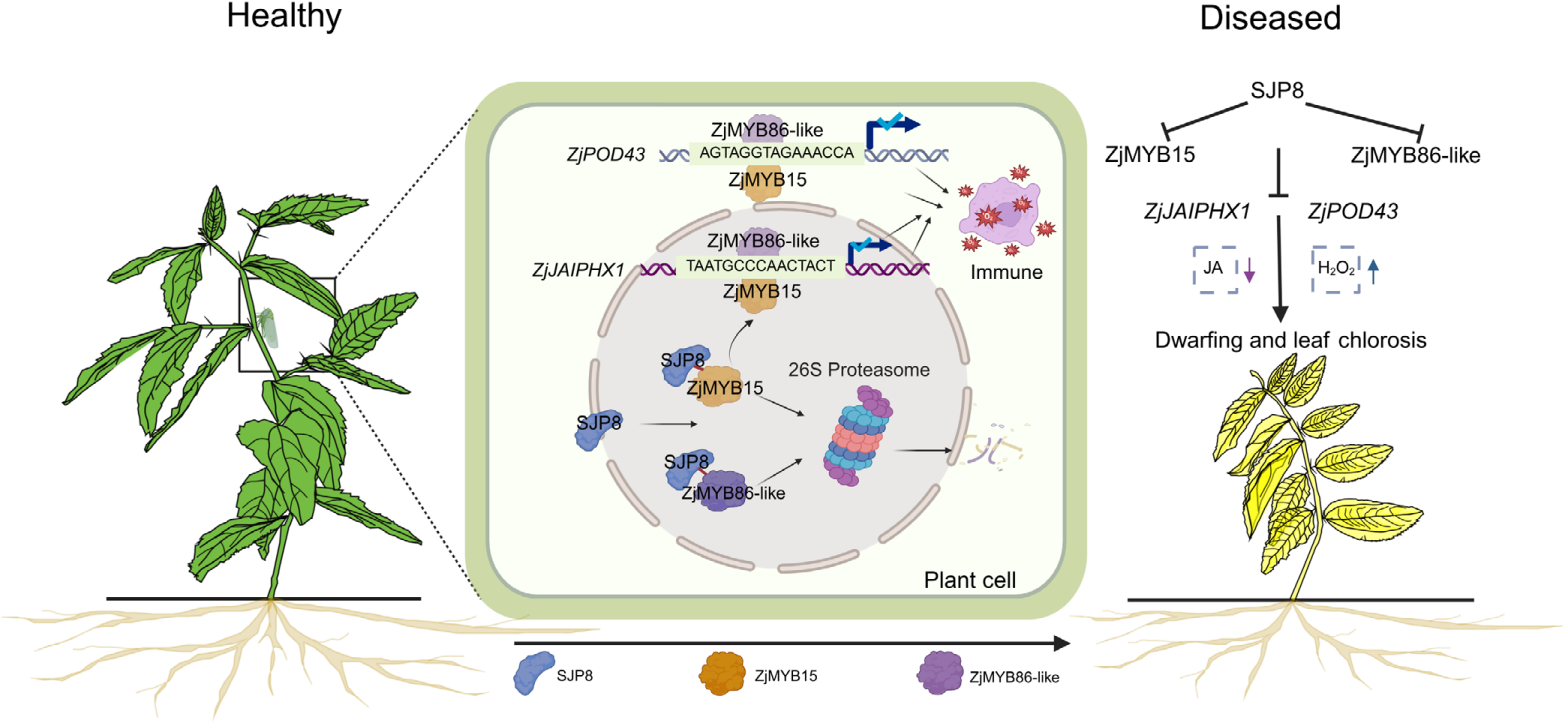
Proposed model for SJP8‐induced dwarfism and leaf chlorosis in transgenic Jingzao 39. SJP8 interacts with the transcription factors ZjMYB15 and ZjMYB86‐like and promotes their degradation via the 26S proteasome pathway. Under normal conditions, ZjMYB15 and ZjMYB86‐like activate the transcription of *ZjJAIPHX1* and *ZjPOD43*. Their degradation leads to downregulation of *ZjJAIPHX1* and *ZjPOD43*, resulting in decreased jasmonic acid (JA) content and increased H_2_O_2_ accumulation. Consequently, plant immunity is compromised and growth and development are disrupted, ultimately causing dwarfism and leaf chlorosis. In the model, blue check marks (✓) and upward arrows (↑) indicate activation, whereas purple downward arrows (↓) indicate suppression.

Based on our experimental findings, we propose a regulatory module, designated SJP8‐ZjMYB15/ZjMYB86‐like‐*ZjJAIPHX1*/*ZjPOD43*, that underlies the dwarfism and chlorosis induced by SJP8 in jujube (Figure 8). In this module, SJP8 interacts with the transcription factors ZjMYB15 and ZjMYB86‐like and promotes their degradation via the 26S proteasome pathway. Under normal conditions, ZjMYB15 and ZjMYB86‐like act as transcriptional activators of *ZjJAIPHX1* and *ZjPOD43*. Their degradation suppresses the expression of *ZjJAIPHX1*, thereby reducing JA levels and downregulates *ZjPOD43*, leading to increased H_2_O_2_ accumulation. This disruption of redox and hormone homeostasis impairs the plant immune system, ultimately causing disease symptoms such as dwarfing and leaf chlorosis. Together, these findings provide new insights into the pathogenic mechanisms of jujube witches' broom and may shed new light on strategies to mitigate this disease in the jujube industry.

## 4 Experimental Procedures

### 4.1 Plant Materials and Growth Conditions

Transgenic *A. thaliana* (Col‐0) and *N*. *benthamiana* plants were generated and grown in a growth chamber under a 16‐h light/8‐h dark photoperiod at 22°C ± 2°C, 60% relative humidity, and a light intensity of 150 μmol m^−2^ s^−1^. For jujube, tissue‐cultured seedlings of transgenic Jingzao 39 (a *Ziziphus* cultivar named after its place of origin) were used. This cultivar is moderately susceptible to JWB disease (Pan et al. 2003; Mu 2025). These seedlings were maintained in a tissue culture room under the same photoperiod and temperature but at 50% relative humidity and a light intensity of 120 μmol m^−2^ s^−1^.

### 4.2 Yeast Secretion Assay for Signal Peptide Function

Full‐length SJP8 (SJP8WP), signal peptide‐only (SJP8SP) and signal peptide‐truncated (SJP8ΔSP) variants were inserted into pSUC2 following EcoRI and XhoI double digestion and transformed into the invertase‐deficient yeast strain YTK12 using the polyethylene glycol/lithium acetate method. Transformants were selected on CMD/−W agar at 30°C for 3–5 days. Positive colonies were streaked onto YPDA, CMD/−W and YPRAA media. Single colonies from YPDA were cultured in CMD liquid medium at 30°C for 24 h, harvested, washed and resuspended in 10% sucrose. An equal volume of 1% TTC was added, and the mixture was incubated at 35°C for 30 min followed by 5 min at room temperature. Colour change was recorded. The experiment was performed twice.

### 4.3 Phylogenetic Analysis

The full‐length amino acid sequence of SJP8 was retrieved from NCBI and used as a query to identify homologous proteins. Eighteen closely related sequences were selected and aligned using ClustalW implemented in MEGA11. A phylogenetic tree was constructed from the aligned sequences using the neighbour‐joining method. The robustness of the tree was assessed by bootstrap analysis with 1000 replicates. The resulting tree was categorised into three distinct clades based on phylogenetic relationships, designated as Class I, Class II and Class III, each highlighted with a distinct colour. In addition, the multiple sequence alignment results were beautified using GenDoc software.

### 4.4 Protein Extraction and Western Blotting

Plant leaves (300 mg) were ground in liquid nitrogen using a grinding apparatus (Jingxin) at 60 Hz for 90 s. The powder was mixed on ice with 500 μL RIPA lysis buffer (Beyotime) containing 1 mM PMSF (Sangon), lysed for 1 h and centrifuged at 12,000 rpm (4°C, 2 min). The supernatant (100 μL) was mixed with an equal volume of 2 × SDS loading buffer, boiled at 100°C for 10 min and stored at −20°C. Proteins were separated by SDS‐PAGE (stacking gel at 80 V for 30 min; separating gel at 110 V for 1 h) and transferred onto PVDF membranes (Bio‐Rad) using a wet transfer system (200 mA, 1.5 h). Membranes were blocked with 5% non‐fat milk (Sangon) in Tris‐buffered saline with Tween 20 (TBST) for 2–4 h at room temperature, incubated with primary antibody (2 h), then with secondary antibody (1 h), with three TBST washes after each incubation. Protein bands were visualised using a chemiluminescent substrate (Polymeric) and imaged with a gel documentation system (Bio‐Rad).

### 4.5 Subcellular Localisation Assay and Microscopic Observation

pBI121‐GFP, pBI121‐*SJP8*‐GFP and the nuclear‐cytoplasmic marker pBI121‐*OsGRX20*‐mCherry were introduced into *Agrobacterium tumefaciens* GV3101. Transformed bacteria were resuspended in infiltration buffer (10 mM MES, 10 mM MgCl_2_, 150 μM acetosyringone, pH 5.6) to OD_600_ 1.0. GFP or *SJP8*‐GFP constructs were mixed 1:1 with the marker and co‐infiltrated into *N. benthamiana* leaves (5–6 weeks old). After 24 h dark incubation, leaves were observed under a laser confocal microscope (Leica) at 48–60 h post‐infiltration. For plasmolysis, leaves were treated with 0.7 M mannitol (Bairige) for 40–50 min before imaging. Three biological replicates were performed. Scale bar = 25 μm. Confocal settings: GFP excitation 488 nm, emission 500–550 nm; mCherry excitation 552 nm, emission 575–625 nm. Empty GFP vector served as negative control. The pBI121‐*OsGRX20*‐mCherry marker localises to the nucleus and cytoplasm (Ning et al. 2018).

### 4.6 Transient Expression Assays

The coding sequence of SJP8 (without signal peptide) was amplified from diseased jujube stem DNA and inserted into pGR107 using seamless cloning (Vazyme) after BamHI digestion (NEB). Empty pGR107 served as a control. Constructs were transformed into *A. tumefaciens* GV3101. Bacterial cultures (OD_600_ = 1.0) were infiltrated into leaves of 4‐week‐old *N. benthamiana*. After 24 h in darkness, plants were grown under a 16‐h light/8‐h dark photoperiod. Phenotypes were recorded at 7, 14 and 21 dpi. For protein detection, 300 mg leaf tissue was frozen in liquid nitrogen; for RNA extraction (Vazyme), 100 mg leaf tissue was collected. Plants with high *SJP8* expression were analysed for JA‐ and H_2_O_2_‐related gene expression.

For vacuum infiltration, *Agrobacterium* harbouring SJP8 or deletion mutants (OD_600_ = 1.0) was used. One‐month‐old, non‐rooted Jingzao 39 plantlets were vacuum‐infiltrated sequentially at 0.045 MPa (40 min) and 0.07 MPa (10 min). After 24 h in darkness, plantlets were transferred to a 16 h light/8 h dark photoperiod for 48 h. Leaf and stem tissues (100 mg each) were harvested separately and frozen. Protein expression was analysed by western blotting, and transcript levels of JA‐ and H_2_O_2_‐related genes by RT‐qPCR. The same procedure was applied to 1‐month‐old, soil‐grown *N. benthamiana* plants, except that all leaves except the top two were removed, and the above‐ground part was inverted and fully submerged during vacuum treatment to target stem tissues. Primers are listed in Table S21.

### 4.7 Bacterial Inoculation Assay

*Pseudomonas syringae* pv. *tomato* DC3000 was streaked from a −80°C glycerol stock onto Luria Bertani (LB)‐rifampicin (25 μg/mL) plates and incubated at 28°C for 48–72 h. A single colony was cultured overnight in LB‐rifampicin at 28°C with shaking, then diluted to mid‐log phase (OD_600_ = 0.6). Cells were harvested (500 *g*, 5 min), washed and resuspended in 10 mM MgCl_2_ to OD_600_ = 0.2 (~1 × 10^8^ CFU/mL). Fully expanded leaves from 4‐ to 6‐week‐old Jingzao 39 plantlets were used. Leaves were photographed before infiltration (0 dpi) as pretreatment controls. At least 10 leaves per line were inoculated. Plants were kept under a 16‐h light/8‐h dark photoperiod. At 5 dpi, phenotypic differences were evident; leaves were photographed and collected for RT‐qPCR and bacterial titration. For bacterial titre quantification, six leaf discs (6 mm diameter) per line were surface‐disinfected (75% ethanol, 10 s), rinsed and homogenised in 500 μL 10 mM MgCl_2_ (28 Hz, 1 min). Serial dilutions were plated on LB‐rifampicin. Colonies were counted after 36–48 h at 28°C and expressed as CFU/cm^2^. The experiment was repeated twice with consistent results.

### 4.8 Y2H Assay

The coding sequence of SJP8 (without signal peptide) was cloned into pENTR‐TOPO (Aidlab) and transferred into pDEST32 (Thermo Fisher Scientific) via Gateway technology to generate the bait. The pDEST32‐*SJP8* construct was transformed into yeast strain AH109 using the polyethylene glycol/lithium acetate method. An *A. thaliana* transcription factor library (Pruneda‐Paz et al. 2014) was used to screen interacting preys. Bait and prey plasmids were co‐transformed into AH109, plated on SD/−Leu−Trp (SD/−LT) medium (Takara) and incubated for 3–5 days. Positive colonies were transferred to SD/−Leu−Trp−His (SD/−LTH) medium containing 10 mM 3‐AT (Sangon) and to SD/−Leu−Trp−His−Ade (SD/−LTHA) medium (Takara), then incubated at 30°C for 3–5 days.

For jujube interaction assays, *SJP8* and its mutants (*SJP8ΔC, SJP8ΔN, SJP8ΔCΔcc*) (without signal peptide) were cloned into pGBKT7 as baits; candidate interacting proteins from Jingzao 39 were cloned into pGADT7 as preys. Bait and prey plasmids were co‐transformed into AH109, plated on SD/−LT medium and incubated for 3–5 days. Positive transformants were transferred to SD/−LTHA medium and incubated at 30°C for 3–5 days. To map interaction domains, single‐base mutation primers were designed and mutant constructs were generated using a Single Base Mutation Kit (Vazyme) by PCR, then cloned into pGBKT7 via homologous recombination. The experiment was performed twice. Primers are listed in Table S21.

### 4.9 Split‐Luciferase Complementation Assay

The coding sequence of *SJP8* (without signal peptide) was cloned into pCAMBIA1300‐Nluc using seamless cloning. Ten *A. thaliana* and seven jujube transcription factors were similarly cloned into pCAMBIA1300‐Cluc. Both constructs were transformed into *A. tumefaciens* GV3101. Bacteria were resuspended in infiltration buffer (10 mM MES, 10 mM MgCl_2_, 150 μM acetosyringone, pH 5.6). Nluc‐ and Cluc‐construct suspensions were mixed 1:1 and co‐infiltrated into tobacco leaves (5–6 leaves/sample). After 24 h in darkness, leaves were kept under normal light for 48–60 h, then treated with 150 μg/mL D‐fluorescein potassium salt (Biorigin) and imaged using a Tanon 5200 Multi Chemiluminescent Imaging System (Tanon).

### 4.10 GST Pull‐Down Assay

The coding sequence of SJP8 (without signal peptide) was cloned into pGEX‐4 T‐1 by EcoRI and seamless cloning (primers in Table S21). AtMYB61 and ZjMYB15 were similarly cloned into pET‐28a. Constructs were transformed into *Escherichia coli* BL21(DE3). GST‐tagged proteins (pGEX‐GST, pGEX‐*SJP8*‐GST) were induced with 1 mM IPTG at 18°C (200 rpm) and purified using GST resin (Yeasen). His‐tagged proteins (pET‐*AtMYB61*‐His, pET‐*ZjMYB15*‐His) were induced with 1 mM IPTG at 16°C (Sangon) and purified using His resin (Yeasen). For pull‐down, GST‐SJP8 or GST alone was incubated with His‐AtMYB61 or His‐ZjMYB15 and 30 μL GST resin at 4°C for 2 h. A 100 μL supernatant aliquot was taken as input. The resin was washed three times with 1 × phosphate‐buffered saline (PBS) (120 rpm, 4°C), then mixed with 60 μL of 2 × SDS loading buffer, boiled at 100°C for 10 min and cooled on ice for 2 min. Samples were analysed by SDS‐PAGE and western blotting.

### 4.11 Co‐IP Assay

The coding sequence of *AtMYB61* was cloned into pCAMBIA1300‐FLAG using HindIII and SalI double digestion followed by seamless cloning. The construct, along with pBI121‐*SJP8* and P19, was transformed into *A. tumefaciens* GV3101. Bacteria were resuspended in infiltration buffer (10 mM MES, 10 mM MgCl_2_, 150 μM acetosyringone, pH 5.6) to OD_600_ = 1.0, mixed at a 1:1:1 ratio and co‐infiltrated into tobacco leaves (6–8 leaves/plant). After 24 h in darkness, plants were grown under normal light for 72 h. Infiltrated leaf tissue (300 mg) was homogenised in 600 μL RIPA lysis buffer (Beyotime) containing 1 mM PMSF (Sangon) and incubated on ice for 1 h. The lysate was centrifuged at 12,000 rpm (4°C, 10 min). A 100 μL aliquot of supernatant was taken as input. The remaining supernatant (500 μL) was incubated with 40 μL anti‐FLAG magnetic beads (Lablead) at 4°C for 50 min with gentle rotation. Beads were washed three times with lysis buffer, then mixed with 60 μL 2 × SDS loading buffer, boiled at 100°C for 10 min and analysed by western blotting to detect GFP‐ and FLAG‐tagged proteins.

### 4.12 RNA‐Seq

Stem samples were collected from 2‐month‐old transgenic jujube (Jingzao 39) and 30‐day‐old T_3_ transgenic *A. thaliana* , with three independent lines for both empty vector control and target gene expression. For each species, 900 mg of stem tissue per line was pooled separately and divided into three aliquots to generate three technical replicates. RNA extraction, library construction and sequencing were performed by Biomarker (Beijing, China). Transcriptome analysis of jujube was conducted using the Jingzao 39 genome as reference, and that of *A. thaliana* using the TAIR10 genome. Raw RNA‐seq data have been deposited in the NCBI under accession numbers PRJNA1163963 (jujube) and PRJNA1163874 ( *A. thaliana* ).

### 4.13 RNA Extraction, RT‐PCR, RT‐qPCR

Stem or leaf samples were collected from 2‐month‐old T_3_ generation transgenic *A. thaliana* (transgenic *N. tabacum* /transgenic *N. benthamiana*/transgenic Jingzao39) for both the control and the experimental treatment groups. RNA extraction was performed using the RNA extraction kit (Vazyme). A total of 2 μg of RNA was used for cDNA synthesis using reverse transcriptase (Vazyme). Subsequently, 1 μL of cDNA was used as a template for qPCR, prepared using the SYBR method (Aidlab). The reactions were performed on a fluorescence‐based quantitative PCR instrument (Thermo Fisher Scientific). The relative expression levels of the target gene were calculated using the 2^−ΔΔ*C*t^ method. The internal reference genes used for normalisation were *AtActin* for *A. thaliana* , *NtActin* for *N. tabacum, NbActin* for *N. benthamiana*, and *ZjActin* for Jingzao39. Primers used for this assay are listed in Table S21.

### 4.14 Plant Tissue Sections and Microscopic Observation

Stem segments (~1 mm) from 2‐month‐old T_3_ transgenic *A. thaliana* or transgenic Jingzao 39 (both control and experimental lines) were embedded in 5% agarose (Biowest) and allowed to solidify. The embedded samples were sectioned and stained with 1% toluidine blue (Servicebio). Tissue structure was examined and documented using a standard light microscope (Leica).

### 4.15 Obtaining Transgenic Plants and Phenotypic Observation

The coding sequence of SJP8 (without signal peptide) was cloned into the pBI121‐GFP vector using XbaI and BamHI double digestion and seamless cloning. The construct was transformed into *A. tumefaciens* GV3101, and bacteria were cultured to an OD_600_ of 0.6–0.8. *A. thaliana* was transformed by the floral dip method, while tobacco and Jingzao 39 jujube were transformed using the leaf disk method. For T_3_ transgenic *A. thaliana* , positive plants were identified and subjected to RT‐qPCR, western blotting and phenotypic observation. For T_1_ transgenic *N. tabacum* , positive plants were similarly identified and analysed. Transgenic Jingzao 39 plants are currently being identified and characterised by RT‐qPCR, western blotting and phenotypic observation.

### 4.16 Measuring Hormone Levels

Leaves (~1 g) were collected from *SJP8*‐overexpressing transgenic plants and null control plants, thoroughly ground and divided into three replicates. Target compounds were separated using a Waters ACQUITY I‐Class ultra‐high performance liquid chromatograph (UHPLC) equipped with an ACQUITY UPLC HSS T3 column (100 × 2.1 mm, 1.8 μm, Waters). Mass spectrometry was performed in multiple reaction monitoring (MRM) mode on a SCIEX QTRAP 6500+ triple quadrupole mass spectrometer fitted with an IonDrive Turbo V ESI source (SCIEX). Hormone levels were quantified by UPLC‐MRM‐MS/MS. The experiment was repeated three times.

### 4.17 Measurement of H_2_O_2_ Content

Leaf tissue (~0.1 g) from each transgenic plant was homogenised in 900 μL of PBS. The homogenate was centrifuged at 12,000 rpm for 10 min, and the supernatant was collected. The H_2_O_2_ concentration was measured in triplicate using a microplate reader (Gene5) at 405 nm following the manufacturer's instructions (Nanjing Jiancheng Bioengineering Institute). The H_2_O_2_ content was calculated using a standard curve. The experiment was repeated twice.

### 4.18 Determination of Lignin Content

Stem segments from transgenic jujube plants were oven‐dried at 80°C to constant weight, ground into powder and passed through a 40‐mesh sieve. Approximately 5 mg of powder was weighed into a 10 mL glass tube. Lignin content was determined using a commercial lignin assay kit (Comin Biotechnology) following the manufacturer's instructions (Zhang, Li, Wei, et al. 2025). Measurements were performed for both transgenic and control groups, with three biological replicates per sample.

### 4.19 In Vivo Degradation Assay

*Agrobacterium tumefaciens* strains carrying pBI121‐GFP, pBI121‐SJP8, pCAMBIA1300‐ZjMYB15, pCAMBIA1300‐ZjMYB86‐like and the silencing suppressor *P19* were cultured to an OD_600_ of ~1.0 and infiltrated into *N. benthamiana* leaves. Plants were kept in darkness for 24 h and then returned to light for 44 h before treatment. First, a 26S proteasome inhibition assay was conducted. At 68 h post‐infiltration, leaves were treated with 50 μM MG132 (Yeasen), 50 μM Bortezomib (Yeasen), or 5% DMSO (control) for 4 h. Total proteins were extracted and analysed by western blotting. Next, to determine whether SJP8 promotes or inhibits target protein degradation, a cycloheximide (CHX) chase assay was performed. At 68 h post‐infiltration, leaves were treated with 200 μM CHX (Biorigin), 200 μM CHX + 50 μM MG132, or 5% DMSO. Samples were collected at 0, 2 and 4 h post‐treatment. Total proteins were extracted, and target protein abundance was detected by western blotting with anti‐actin as a loading control. Band intensities were quantified using ImageJ and normalised to actin, and degradation curves were generated.

### 4.20 DAP‐Seq

Approximately 5 g of wild‐type Jingzao 39 tissue‐cultured seedlings (including stems and leaves) were harvested, frozen in liquid nitrogen and sent to Bluecape (Hebei, China) for total plant DNA extraction. The coding sequence of *ZjMYB15* was cloned into the pET‐28a‐His expression vector via homologous recombination. The recombinant protein was expressed in *E. coli* BL21(DE3) and purified using a Ni‐NTA affinity column. A DNA affinity purification library was constructed using the purified protein. Biotinylated DNA probes were used to capture protein–DNA complexes, which were enriched with streptavidin‐coated magnetic beads. Captured DNA fragments were sequenced and analysed bioinformatically. The experiment was performed following the protocol described previously (Bartlett et al. 2017).

### 4.21 Y1H Assay

The promoter fragment was inserted into the pLacZi vector using KpnI and homologous recombination, and the coding sequence of *ZjMYB15* was inserted into the pB42AD vector using EcoRI and homologous recombination. The pB42AD‐transcription factor and pLacZi‐promoter constructs were co‐transformed into the yeast strain EGY48 (Coolabo). Positive controls included co‐transformation with pB42AD‐*53* and pLacZi‐*P53*; negative controls included co‐transformation with empty pB42AD and empty pLacZi vectors. All transformants were plated on SD/−Leu−Ura (SD/−LU) solid medium (Bairige) and incubated at 30°C for 3–5 days. Single colonies were then transferred to SD/−LU medium containing X‐gal and incubated at 30°C for another 3–5 days. Colonies were photographed for documentation. The experiment was repeated twice.

### 4.22 Dual Luciferase Reporter Assay

Promoters of candidate target genes identified by DAP‐seq were cloned into the pGreen II 0800 vector, and the coding sequence of *ZjMYB15* was cloned into the pGreen II 62‐SK vector. Both constructs were transformed into *A. tumefaciens* GV3101. Positive monoclonal colonies were selected and cultured to an OD_600_ of approximately 1.0, then co‐infiltrated into tobacco leaves. After infiltration, leaves were kept in darkness for 24 h, then transferred to normal light for 48–72 h. Fluorescence signals were observed using a protein imager (Tanon). For dual‐luciferase assays, approximately 0.1 g of infiltrated leaf tissue was collected, ground with steel beads and lysed in 100 μL of lysis buffer. The lysate was centrifuged, and the supernatant was collected in triplicate. Firefly and Renilla luciferase activities were measured using the dual‐luciferase reporter assay system (Promega) with a luminometer (Yeasen).

### 4.23 EMSA

The full‐length coding sequence of *ZjMYB86‐like* (stop codon excluded) was amplified and cloned into the pMAL‐c5X‐MBP expression vector via homologous recombination. Recombinant protein expression was induced in *E. coli* BL21(DE3) with 1 mM IPTG at 16°C and 200 rpm. The MBP‐tagged ZjMYB86‐like fusion protein was affinity‐purified using MBP‐specific resin (Yeasen). Protein purified from cells transformed with the empty pMAL‐c5X‐MBP vector (MBP only) served as the negative control. Biotin‐labelled EMSA probes, along with unlabelled (cold) competitor and mutant probes, were synthesised (Ruibo Biotech). Binding reactions containing purified protein (ZjMYB86‐like‐MBP or MBP‐only control), biotin‐labelled probe and competitor probes were assembled following the EMSA kit protocol (Beyotime). Protein–DNA complexes were resolved by native PAGE and transferred to nitrocellulose membranes (Beyotime). Biotin‐labelled DNA was detected using streptavidin‐HRP (Beyotime). Protein‐DNA binding interactions were visualised by chemiluminescence imaging using a gel documentation system (Bio‐Rad). EMSA verification of ZjMYB15 binding to the conserved promoter motifs was performed using the same protocol.

### 4.24 Statistical Analysis

Data analysis in this study was conducted using Excel and GraphPad Prism v. 9.5.1. Root length measurements for transgenic *A. thaliana* , as well as cell length and width of plant tissue sections and leaf area of transgenic tobacco, were obtained using ImageJ software. Statistical significance was assessed with a *p*‐value threshold of < 0.05, using either Student's *t*‐test or one‐way ANOVA performed in GraphPad Prism v. 9.5.1. All experiments were conducted independently at least three times.

## Author Contributions

**Ying Yang:** conceptualization, data curation, formal analysis, investigation, methodology, writing – original draft, writing – review and editing, validation, visualization. **Haizhen Nie:** conceptualization, data curation, formal analysis, investigation, methodology. **Weikai Chen:** conceptualization, data curation, formal analysis, investigation, validation. **Yaru Xu:** data curation, formal analysis, investigation, validation. **Bo Wu:** formal analysis, investigation, visualization. **Ling Ma:** formal analysis, investigation, visualization. **Zhonghua Liu:** visualization, funding acquisition, writing – review and editing. **Xiaoming Pang:** conceptualization, funding acquisition, supervision, writing – review and editing. All authors have read and agreed to the published version of the manuscript.

## Funding

This work was supported by the National Key R&D Program of China (2024YFD2200600), the Hebei Province Academy of Sciences Key Cooperative Unit, research on jujube witches' broom disease pathogen, rhizosphere microorganisms and the interactions and pathogenic mechanisms with jujubes.

## Conflicts of Interest

The authors declare no conflicts of interest.

## Supporting information


**Figure S1:** SJP8 is a key effector protein of ‘*Candidatus* Phytoplasma ziziphi’.

**Figure S2:** Plasmolysis experiments verifying the subcellular localisation of SJP8 in *Nicotiana benthamiana*.

**Figure S3:** Reverse transcription‐quantitative PCR analysis of H_2_O_2_‐related gene expression in *Nicotiana benthamiana* leaves transiently overexpressing *SJP8* at 7 days post‐infiltration.

**Figure S4:** Comparison of root systems in *SJP8*‐overexpressing and empty vector control transgenic Jingzao 39 plants after 28 days of rooting.

**Figure S5:** Detection of H_2_O_2_ levels in leaves of field‐grown jujube plants with varying degrees of jujube witches' broom phytoplasma infection.

**Figure S6:** Overexpression of *SJP8* induces dwarfing in transgenic *Nicotiana tabacum* .

**Figure S7:** Overexpression of *SJP8* induces dwarfism in transgenic *Arabidopsis thaliana*.

**Figure S8:** Validation of randomly selected differentially expressed genes from transcriptome analysis by reverse transcription‐quantitative PCR.

**Figure S9:** Split‐luciferase assays validate the interaction of SJP8 with five out of 10 candidate *Arabidopsis thaliana* transcription factors.

**Figure S10:** Reverse transcription‐quantitative PCR analysis of gene expression for the five interacting proteins in stem segments of T_3_ generation *Arabidopsis thaliana* .

**Figure S11:** Phylogenetic identification of *Arabidopsis thaliana* transcription factor homologues in Jingzao 39 based on gene family analysis.

**Figure S12:** Yeast two‐hybrid and split‐luciferase assays validating the interaction of SJP8 with jujube homologues of selected *Arabidopsis thaliana* transcription factors.

**Figure S13:** Degradation curves of AtMYB61‐FLAG.

**Figure S14:** Interaction between SJP8 deletion mutants and ZjMYB15 and ZjMYB86‐like.

**Figure S15:** Expression analysis of jasmonic acid‐ and H_2_O_2_‐related genes Jingzao39 and *Nicotiana benthamiana* leaves transiently overexpressing *SJP8* and its deletion mutants.

**Figure S16:** Verification of *ZjMYB15* and *ZjMYB86‐like* overexpression and RNAi transgenic Jingzao 39 lines.

**Figure S17:** ZjMYB15 and ZjMYB86‐like positively regulate shoot growth in Jingzao 39.

**Figure S18:** Expression of *ZjLOX2* and *ZjCuAOβ* in *ZjMYB15* and *ZjMYB86‐like* transgenic lines.

**Figure S19:** *ZjLOX2* expression in *ZjMYB15* and *ZjMYB86‐like* transgenic lines in response to *Pseudomonas syringae* pv. *tomato* DC3000 infection.

**Figure S20:** Quantification of ZjMYB15 and ZjMYB86‐like protein levels upon proteasome inhibition.

**Figure S21:** Degradation curves of ZjMYB15‐FLAG and ZjMYB86‐like‐FLAG.

**Figure S22:** Subcellular localisation of ZjPOD43 and ZjJAIPHX1 in *Nicotiana benthamiana*.

**Figure S23:** ZjMYB15 and ZjMYB86‐like activate transcription of *ZjJAIPHX1* and *ZjPOD43*.

**Figure S24:** Reverse transcription‐quantitative PCR analysis of *JAIPHX1* and *POD43* expression in healthy, infected and *SJP8*‐overexpressing transgenic plants.

**Figure S25:** Conservation analysis of the ZjMYB15‐binding motif.

**Table S1:** Candidate effector proteins predicted from the ‘*Candidatus* Phytoplasma ziziphi’ genome.

**Table S2:** RNA‐seq expression values (FPKM) for all genes in *SJP8*‐overexpressing Jingzao 39.

**Table S3:** RNA‐seq expression values (FPKM) for all genes in *SJP8*‐overexpressing *Arabidopsis thaliana* .

**Table S4:** Significantly enriched GO biological processes in *SJP8*‐overexpressing Jingzao 39.

**Table S5:** Significantly enriched GO biological processes in *SJP8*‐overexpressing *Arabidopsis thaliana* .

**Table S6:** Significantly enriched KEGG pathways in *SJP8*‐overexpressing Jingzao 39.

**Table S7:** Significantly enriched KEGG pathways in *SJP8*‐overexpressing *Arabidopsis thaliana* .

**Table S8:** Heatmap of lignin‐related gene expression in the transcriptome of *SJP8*‐overexpressing Jingzao 39.

**Table S9:** Heatmap of lignin‐related gene expression in the transcriptome of *SJP8*‐overexpressing *Arabidopsis thaliana* .

**Table S10:** Heatmap of jasmonic acid‐related gene expression in the transcriptome of *SJP8*‐overexpressing Jingzao 39.

**Table S11:** Heatmap of jasmonic acid‐related gene expression in the transcriptome of *SJP8*‐overexpressing *Arabidopsis thaliana* .

**Table S12:** Heatmap of hydrogen peroxide‐related gene expression in the transcriptome of *SJP8*‐overexpressing Jingzao 39.

**Table S13:** Heatmap of hydrogen peroxide‐related gene expression in the transcriptome of *SJP8*‐overexpressing *Arabidopsis thaliana* .

**Table S14:** *Arabidopsis thaliana* transcription factors identified as SJP8 interactors in yeast two‐hybrid assay primary screening.

**Table S15:** AtHSFA1D, AtBBX27, AtEDT1 and AtbHLH155 and AtMYB61 homologous transcription factors in Jingzao39 identified by phylogenetic analysis.

**Table S16:** DNA affinity purification (DAP)‐seq peak annotations for ZjMYB15 target promoters (biological replicates).

**Table S17:** ZjMYB15 target promoter details for two key immune‐related genes identified by DNA affinity purification (DAP)‐seq.

**Table S18:** The *ZjJAIPHX1* and *ZjPOD43* promoter sequences.

**Table S19:** Clustering heatmap of *JAIHPX1* homologues and jasmonic acid‐related pathway genes in *SJP8*‐overexpressing transgenic plants.

**Table S20:** Clustering heatmap of *POD43* homologues and H_2_O_2_‐related pathway genes in *SJP8*‐overexpressing transgenic plants.

**Table S21:** Primer sequences used in this study.

## Data Availability

Sequence data in this study can be found in NCBI (https://www.ncbi.nlm.nih.gov/) under the following accession number: SJP8 (Zaofeng1, JWB90, AYJ01076.1), AtGATA9 (NM_119442.3), AtGATA5 (NM_126030.6), AtCRF1 (NM_117184.4), AtMYB61 (NM_100825.5), AtHSFA1D (NM_102966.4), AtMYB40 (NM_121438.3), AtBBX27 (NM_001334353.1/NM_105490.4), AtAGL49 (NM_104696.2), AtEDT1 (NM_105996.4), AtBHLH155 (NM_128684.5), ZjMYB86‐like (XP_015872669.2), ZjMYB15 (XP_015880997.3), ZjMYB86 (XP_048323715.2), ZjEOBI (XP_015878099.2), ZjMYB61 (KAH7528412.1), ZjMYB83 (XP_015881673.3), ZjMYB46 (XP_015875881.3), *ZjJAIPHX1* (*Zj10G000470*/XP_048318134.1), *ZjPOD43* (*Zj09G014410/*XP_015892998.1). All other relevant data can be found within the manuscript and its supporting materials.
