# Supplementary material for: Phytoplasma Effector SJP8 Suppresses Host Immunity by Promoting the Degradation of ZjMYB15 and ZjMYB86‐like to Perturb Jasmonic Acid and Hydrogen Peroxide Homeostasis in Jujube
Source: Mol Plant Pathol. 2026 Jul 10;27(7):e70315. doi: 10.1111/mpp.70315 (PMC13351939; doi:10.1111/mpp.70315)
Supplement: Supplementary file 1 — Figure S1: SJP8 is a key effector protein of ‘Candidatus Phytoplasma ziziphi’. [file MPP-27-e70315-s042.docx]

**Figure S1 |** SJP8 is a key effector protein of ‘*Ca*. P. ziziphi’. (a) Annotation pipeline for identifying JWB phytoplasma effector proteins. (b) Detailed information of 15 effector proteins selected for their high expression in jujube and low expression in leafhoppers. SJP8 is highlighted in bold. In the “NLS” column, “+” indicates the presence of a nuclear localization signal, while “-” indicates its absence. In the “Protein ID” column, “-” indicates that no ID is available. The “Other names” column lists effector protein names reported in previous studies (Deng et al., 2021; Chen et al., 2022; Yang et al., 2025). (c) Homology search of SJP8 using NCBI BLASTp after removal of the signal peptide. (d) Gene expression levels of SJP8 in infected ‘Suanzao’ (a variety of jujube) branches and leafhoppers. Statistical significance was determined using Student’s t-test (**p* < 0.05).

**References**

Chen, P., L. Chen, X. Ye, B. Tan, X. Zheng, J. Cheng, W. Wang, Q. Yang, Y. Zhang, J. Li, and J. Feng. 2022. “Phytoplasma effector Zaofeng6 induces shoot proliferation by decreasing the expression of ZjTCP7 in *Ziziphus jujuba*.” *Horticulture Research* 9: uhab032.

Deng, M., F. Ma, X. Zhang, J. Huang, J. Yang, M. Chen, J. Zhou, Q. Sun, and J. Sun. 2021. “Genome-wide identification of jujube witches’ broom phytoplasma effectors revealed the role of SJP3 in inducing phyllody.” *Scientia Horticulturae* 290: 110548.

Yang, S., A.H. Lovelace, Y. Yuan, et al. 2025. “A Witches’ Broom Phytoplasma Effector Induces Stunting by Stabilizing a bHLH Transcription Factor in *Ziziphus jujuba* Plants.” *New Phytologist* 247: 249-264.
