# Supplementary material for: Phytoplasma Effector SJP8 Suppresses Host Immunity by Promoting the Degradation of ZjMYB15 and ZjMYB86‐like to Perturb Jasmonic Acid and Hydrogen Peroxide Homeostasis in Jujube
Source: Mol Plant Pathol. 2026 Jul 10;27(7):e70315. doi: 10.1111/mpp.70315 (PMC13351939; doi:10.1111/mpp.70315)
Supplement: Supplementary file 2 — Figure S2: Plasmolysis experiments verifying the subcellular localisation of SJP8 in Nicotiana benthamiana. [file MPP-27-e70315-s017.docx]

**Figure S2 |** Plasmolysis experiments verifying the subcellular localization of SJP8 in *N. benthamiana*. An empty GFP vector served as a control. The pBI121-*OsGRX20*-mCherry construct (mCherry fluorescence) served as a nuclear-cytoplasmic co-localization marker, as *OsGRX20* has been reported to localize to both the nucleus and cytoplasm (Ning et al., 2018). Scale bar = 50 µm.

**Reference**

Ning, X., Y. Sun, C. Wang, W. Zhang, M. Sun, H. Hu, J. Liu, and L. Yang. 2018. “A rice CPYC-Type Glutaredoxin OsGRX20 in protection against bacterial blight, Methyl Viologen and salt stresses.” *Frontiers in Plant Science* 9: 111.
