# Supplementary material for: Phytoplasma Effector SJP8 Suppresses Host Immunity by Promoting the Degradation of ZjMYB15 and ZjMYB86‐like to Perturb Jasmonic Acid and Hydrogen Peroxide Homeostasis in Jujube
Source: Mol Plant Pathol. 2026 Jul 10;27(7):e70315. doi: 10.1111/mpp.70315 (PMC13351939; doi:10.1111/mpp.70315)
Supplement: Supplementary file 3 — Figure S3: Reverse transcription‐quantitative PCR analysis of H2O2‐related gene expression in Nicotiana benthamiana leaves transiently overexpressing SJP8 at 7 days post‐infiltration. [file MPP-27-e70315-s004.docx]

**Figure S3 |** QRT-PCR analysis of H₂O₂-related gene expression in *N. benthamiana* leaves transiently overexpressing SJP8 at 7 dpi. (a) Expression levels of H₂O₂ production-related genes (*NbRbohB*, *NbRbohD*, *NbRbohF*, and *NbCuAOβ*). (b) Expression levels of H₂O₂ scavenging‑related genes (*NbCAT1* and *NbAPX2*). (c) Expression levels of H₂O₂ signal transduction‑related genes (*NbKIN10*, *NbOXI1*, and *NbSPCH*). The GFP‑expressing group served as the control for all panels. For panels (a)-(c), statistical analysis was performed using one‑way ANOVA with Tukey’s test. Error bars represent the SD of three technical replicates. Significance levels are indicated as follows: **p* < 0.05, ***p* < 0.01, ***p < 0.001, *****p* < 0.0001. All experiments were repeated three times with consistent results. *NbActin* was used as an internal reference gene.
