# Supplementary material for: Phytoplasma Effector SJP8 Suppresses Host Immunity by Promoting the Degradation of ZjMYB15 and ZjMYB86‐like to Perturb Jasmonic Acid and Hydrogen Peroxide Homeostasis in Jujube
Source: Mol Plant Pathol. 2026 Jul 10;27(7):e70315. doi: 10.1111/mpp.70315 (PMC13351939; doi:10.1111/mpp.70315)
Supplement: Supplementary file 4 — Figure S4: Comparison of root systems in SJP8‐overexpressing and empty vector control transgenic Jingzao 39 plants after 28 days of rooting. [file MPP-27-e70315-s025.docx]

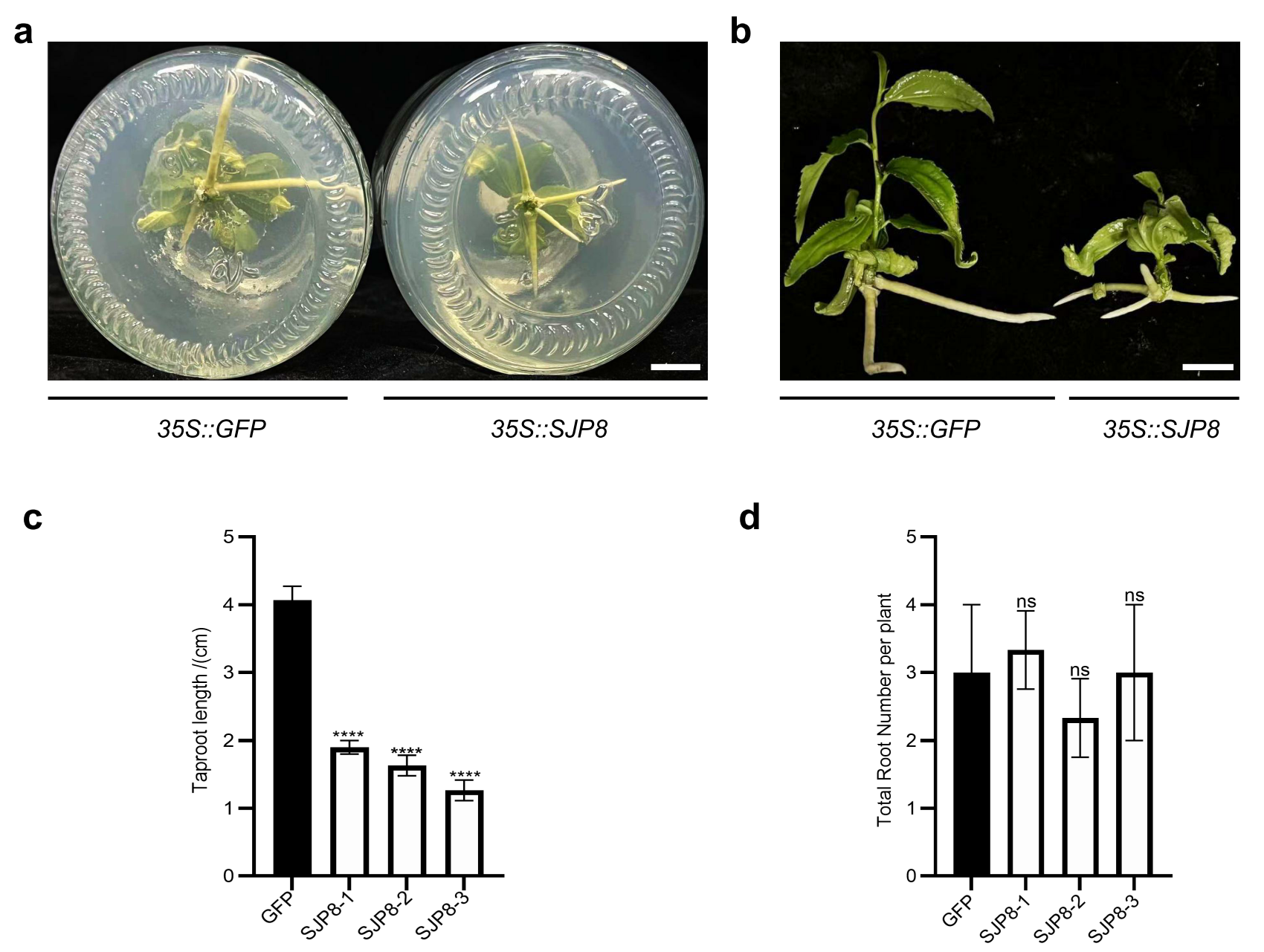


**Figure S4 |** Comparison of root systems in SJP8‑overexpressing and empty vector control transgenic ‘Jingzao 39’ plants after 28 days of rooting. (a) Root phenotypes of SJP8-overexpressing and control lines grown in tissue culture flasks. Scale bar = 1 cm. (b) Close-up comparison of root systems between SJP8-overexpressing and control lines. Scale bar = 1 cm. (c) Quantitative comparison of primary root length between SJP8-overexpressing and EV control lines. Data are presented as mean ± SD (n = 3 biological replicates). The asterisk indicates a statistically significant difference as determined by one-way ANOVA (p < 0.05). (d) Quantitative comparison of total root number between SJP8‑overexpressing and EV control lines. Data are presented as mean ± SD (n = 3 biological replicates). ns, not significant, *****p* < 0.0001.
