# Supplementary material for: Phytoplasma Effector SJP8 Suppresses Host Immunity by Promoting the Degradation of ZjMYB15 and ZjMYB86‐like to Perturb Jasmonic Acid and Hydrogen Peroxide Homeostasis in Jujube
Source: Mol Plant Pathol. 2026 Jul 10;27(7):e70315. doi: 10.1111/mpp.70315 (PMC13351939; doi:10.1111/mpp.70315)
Supplement: Supplementary file 5 — Figure S5: Detection of H2O2 levels in leaves of field‐grown jujube plants with varying degrees of jujube witches' broom phytoplasma infection. [file MPP-27-e70315-s039.docx]

**Figure S5 |** Detection of H₂O₂ levels in leaves of field-grown jujube plants with varying degrees of JWB phytoplasma infection. (a) Leaf samples from a healthy plant and plants with graded JWB infection severity (slight, moderate, and severe), classified based on the extent of chlorosis. Scale bar = 1 cm. (b) Quantification of H₂O₂ levels in the leaf samples shown in (a). Data are presented as mean ± SD of three technical replicates. Statistical significance was determined by one‑way ANOVA (**p* < 0.05, ***p* < 0.01.).
