# Supplementary material for: Phytoplasma Effector SJP8 Suppresses Host Immunity by Promoting the Degradation of ZjMYB15 and ZjMYB86‐like to Perturb Jasmonic Acid and Hydrogen Peroxide Homeostasis in Jujube
Source: Mol Plant Pathol. 2026 Jul 10;27(7):e70315. doi: 10.1111/mpp.70315 (PMC13351939; doi:10.1111/mpp.70315)
Supplement: Supplementary file 6 — Figure S6: Overexpression of SJP8 induces dwarfing in transgenic Nicotiana tabacum . [file MPP-27-e70315-s019.docx]

**Figure S6 |** Overexpression of SJP8 induces dwarfism in transgenic *N. tabacum*. (a) Growth status of T1 generation transgenic *N. tabacum* after one month. Scale bar = 10 cm. (b) Western blot analysis of SJP8 protein expression in T1 transgenic *N. tabacum*. The large subunit of ribulose‑1,5‑bisphosphate carboxylase/oxygenase (Rubisco), visualized by Coomassie blue staining, served as a loading control. Molecular weight markers (kDa) are indicated on the right. (c) Plant height measurement of T1 transgenic *N. tabacum* after one month of growth (n = 3 plants per line). (d) Lignin content in transgenic plants. (e) Statistical analysis of leaf number in T1 transgenic *N. tabacum*. (f) Leaf size observation. Scale bar = 10 cm. (g) Statistical analysis of leaf area. Red solid triangles indicate SJP8‑overexpressing plants; black solid circles indicate empty vector control plants. (h-j) Relative expression levels of JA biosynthesis (h), metabolism (i), and signal transduction (j) genes in leaves of one‑month‑old *N. tabacum* plants overexpressing SJP8, determined by qRT‑PCR. (k-m) Relative expression levels of H₂O₂ production (k), scavenging (l), and signal transduction (m) genes in the same samples, determined by qRT‑PCR. For panels (c), (e), (h-j), and (k-m), statistical analysis was performed using one-way ANOVA with Tukey’s test. For panel (d), Student’s t-test was used. Error bars represent the SD of three technical replicates. Significance levels are indiacted as follows: ns, not significant *p* > 0.05, **p* < 0.05, ***p* < 0.01, ****p* < 0.001, *****p* < 0.0001. All experiments were repeated three times with consistent results. NtActin was used as an internal reference gene.
