# Supplementary material for: Phytoplasma Effector SJP8 Suppresses Host Immunity by Promoting the Degradation of ZjMYB15 and ZjMYB86‐like to Perturb Jasmonic Acid and Hydrogen Peroxide Homeostasis in Jujube
Source: Mol Plant Pathol. 2026 Jul 10;27(7):e70315. doi: 10.1111/mpp.70315 (PMC13351939; doi:10.1111/mpp.70315)
Supplement: Supplementary file 7 — Figure S7: Overexpression of SJP8 induces dwarfism in transgenic Arabidopsis thaliana. [file MPP-27-e70315-s001.docx]

**Figure S7** | Overexpression of SJP8 induces dwarfism in transgenic *A. thaliana.* (a) Phenotypes of T1 generation transgenic *A. thaliana*. Scale bar = 10 cm. (b) Root length of T3 generation plants after two weeks of growth. Scale bar = 1 cm. (c) Western blot analysis of SJP8 protein expression in T3 transgenic lines. Coomassie‑stained Rubisco large subunit served as a loading control. Molecular weight markers (kDa) are indicated on the right. (d) Quantification of root length in the transgenic lines. (e) Phenotypes of T3 generation plants. Scale bar = 10 cm. (f) Plant height of T3 transgenic lines after two weeks of growth. Scale bar = 10 cm. (g) Quantification of plant height. (h) Quantification of rosette leaf number. (i) Cross‑sections of stems stained with methylene blue. Scale bar = 100 µm. (j) Quantification of cell length and width in stem cross‑sections. (k) Quantification of lignin content in stem segments of T3 transgenic lines. (l) QRT‑PCR analysis of JA‑related genes in leaves of SJP8‑overexpressing lines, including biosynthesis (*AtLOX2*, *AtLOX4*), metabolism (*AtJAO4*, *AtJMT2*), and signal transduction (*AtCOI1*, *AtPDF1.2*, *AtVSP1*). (m) QRT‑PCR analysis of H₂O₂‑related genes, including production (*AtRbohD*, *AtCuAOβ*), scavenging (*AtPER12*, *AtPER56*, *AtPER66*), and signal transduction genes (*AtOXI1*, *AtSPCH*). GFP‑expressing plants served as controls in (l) and (m). *AtActin* was used as an internal reference. For panels (d), (g), (h), (l), and (m), statistical significance was assessed using one‑way ANOVA. For (j) and (k), Student’s t‑test was used. Data are presented as mean ± SD of three biological replicates. Significance levels are indicated as follows: **p* < 0.05, ***p* < 0.01, ****p* < 0.001, *****p* < 0.0001. All experiments were repeated three times with consistent results.
