# Supplementary material for: Phytoplasma Effector SJP8 Suppresses Host Immunity by Promoting the Degradation of ZjMYB15 and ZjMYB86‐like to Perturb Jasmonic Acid and Hydrogen Peroxide Homeostasis in Jujube
Source: Mol Plant Pathol. 2026 Jul 10;27(7):e70315. doi: 10.1111/mpp.70315 (PMC13351939; doi:10.1111/mpp.70315)
Supplement: Supplementary file 8 — Figure S8: Validation of randomly selected differentially expressed genes from transcriptome analysis by reverse transcription‐quantitative PCR. [file MPP-27-e70315-s028.docx]

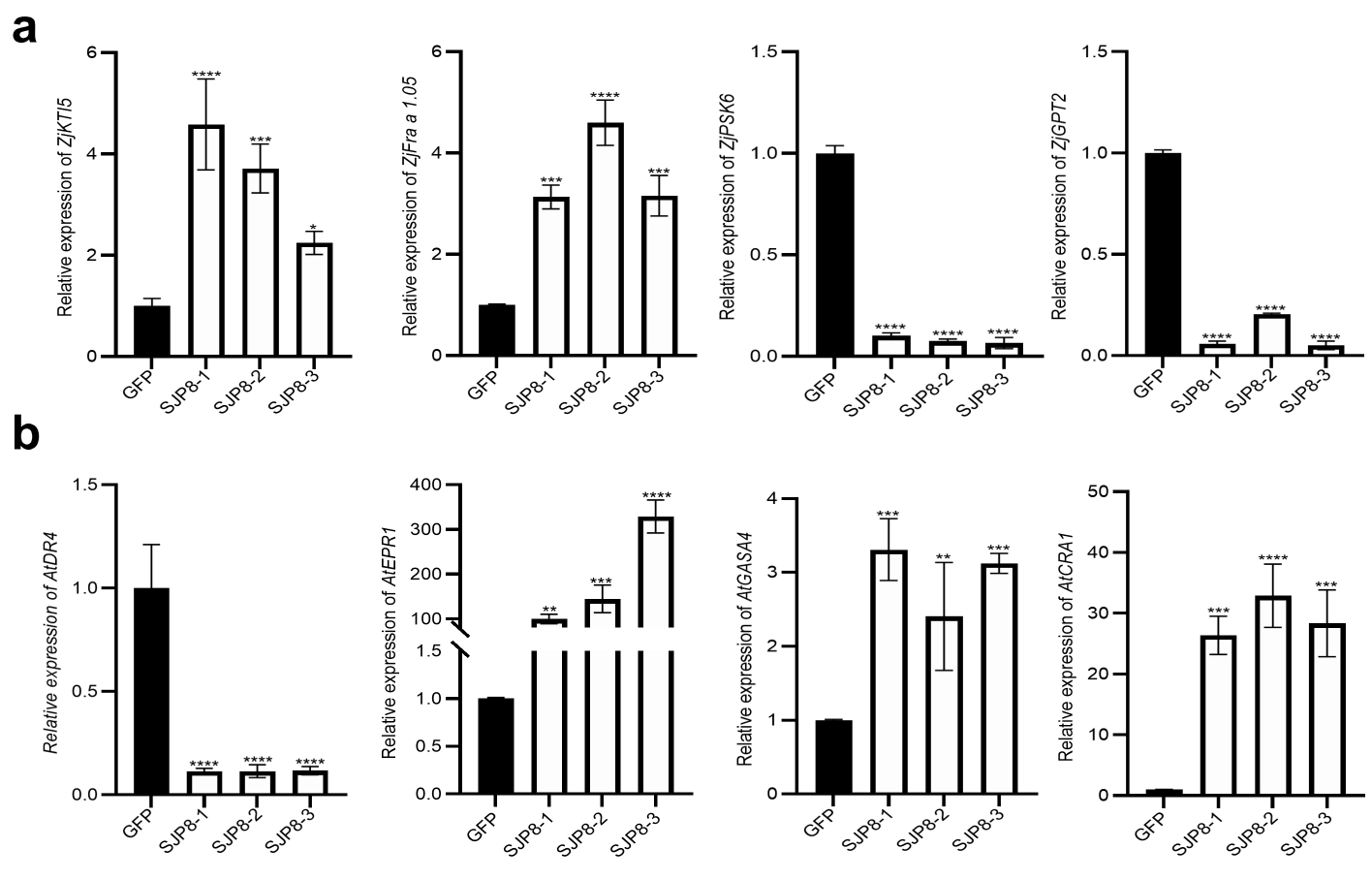


**Figure S8** **|** Validation of randomly selected differentially expressed genes from transcriptome analysis by qRT-PCR. (a) Validation of randomly selected genes in *Z. jujuba* (‘Jingzao 39’). *ZjActin* was used as an internal reference gene. (b) Validation of randomly selected genes in *A. thaliana*. *AtActin* was used as an internal reference gene. Data are presented as mean ± SD of three technical replicates. Asterisks indicate statistically significant differences as determined by one-way ANOVA (**p* < 0.05, ***p* < 0.01, ****p* < 0.001, *****p* < 0.0001).
