# Supplementary material for: Phytoplasma Effector SJP8 Suppresses Host Immunity by Promoting the Degradation of ZjMYB15 and ZjMYB86‐like to Perturb Jasmonic Acid and Hydrogen Peroxide Homeostasis in Jujube
Source: Mol Plant Pathol. 2026 Jul 10;27(7):e70315. doi: 10.1111/mpp.70315 (PMC13351939; doi:10.1111/mpp.70315)
Supplement: Supplementary file 9 — Figure S9: Split‐luciferase assays validate the interaction of SJP8 with five out of 10 candidate Arabidopsis thaliana transcription factors. [file MPP-27-e70315-s036.docx]

**Figure S9 |** Split‑LUC assays validate the interaction of SJP8 with five out of ten candidate *Arabidopsis* transcription factors. (a) Schematic of the split‑luciferase complementation (Split‑LUC) constructs. (b) Split‑LUC assays confirm that SJP8 interacts with AtbHLH155, AtHSF1D, AtMYB61, AtBBX27, and AtEDT1. Cluc and Nluc empty vectors served as controls.
