# Supplementary material for: Phytoplasma Effector SJP8 Suppresses Host Immunity by Promoting the Degradation of ZjMYB15 and ZjMYB86‐like to Perturb Jasmonic Acid and Hydrogen Peroxide Homeostasis in Jujube
Source: Mol Plant Pathol. 2026 Jul 10;27(7):e70315. doi: 10.1111/mpp.70315 (PMC13351939; doi:10.1111/mpp.70315)
Supplement: Supplementary file 10 — Figure S10: Reverse transcription‐quantitative PCR analysis of gene expression for the five interacting proteins in stem segments of T3 generation Arabidopsis thaliana . [file MPP-27-e70315-s012.docx]

**Figure S10 |** QRT-PCR analysis of gene expression for the five interacting proteins in stem segments of T3 generation *A. thaliana*. Data were normalized to the healthy control group and are presented as mean ± SD (n = 3). Statistical analysis was performed using one-way ANOVA; significance was defined as ***p* < 0.01, ****p* < 0.001, *****p* < 0.0001. AtActin was used as an internal reference gene.
