# Supplementary material for: Phytoplasma Effector SJP8 Suppresses Host Immunity by Promoting the Degradation of ZjMYB15 and ZjMYB86‐like to Perturb Jasmonic Acid and Hydrogen Peroxide Homeostasis in Jujube
Source: Mol Plant Pathol. 2026 Jul 10;27(7):e70315. doi: 10.1111/mpp.70315 (PMC13351939; doi:10.1111/mpp.70315)
Supplement: Supplementary file 11 — Figure S11: Phylogenetic identification of Arabidopsis thaliana transcription factor homologues in Jingzao 39 based on gene family analysis. [file MPP-27-e70315-s010.docx]

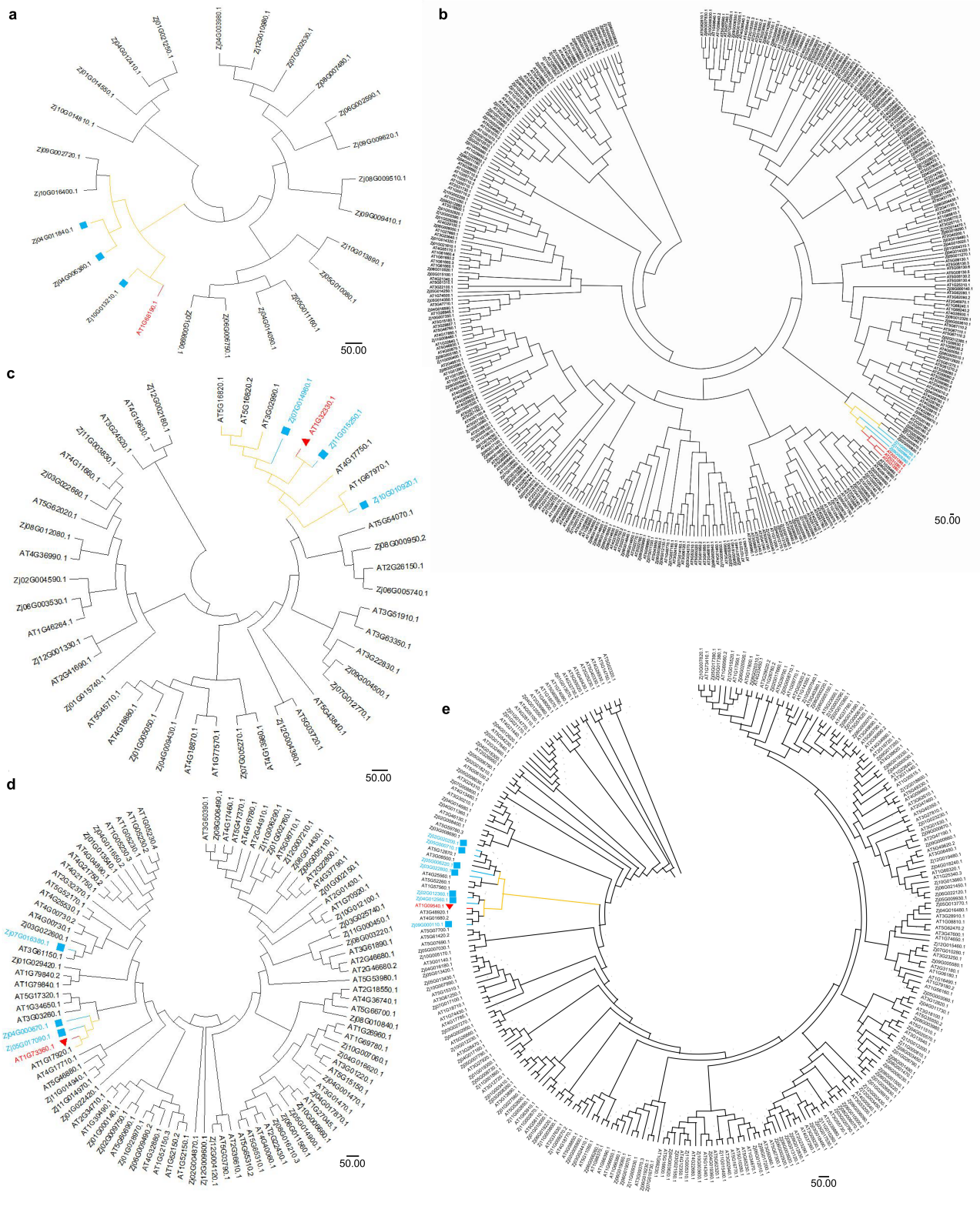


**Figure S11 |** Phylogenetic identification of *A. thaliana* transcription factor homologs in ‘Jingzao 39’ based on gene family analysis. (a-e) Gene family analysis of ‘Jingzao 39’ genes homologous to AtBBX27 (AT1G68190.1), AtHSFA1D (AT1G32330.1), AtEDT1 (AT1G73360.1), AtbHLH155 (AT2G31280.1, AT2G31280.2, AT2G31280.3), and AtMYB61 (AT1G09540.1). Red triangles indicate *A. thaliana* transcription factors, and blue squares indicate ‘Jingzao 39’ transcription factors.
