# Supplementary material for: Phytoplasma Effector SJP8 Suppresses Host Immunity by Promoting the Degradation of ZjMYB15 and ZjMYB86‐like to Perturb Jasmonic Acid and Hydrogen Peroxide Homeostasis in Jujube
Source: Mol Plant Pathol. 2026 Jul 10;27(7):e70315. doi: 10.1111/mpp.70315 (PMC13351939; doi:10.1111/mpp.70315)
Supplement: Supplementary file 12 — Figure S12: Yeast two‐hybrid and split‐luciferase assays validating the interaction of SJP8 with jujube homologues of selected Arabidopsis thaliana transcription factors. [file MPP-27-e70315-s026.docx]

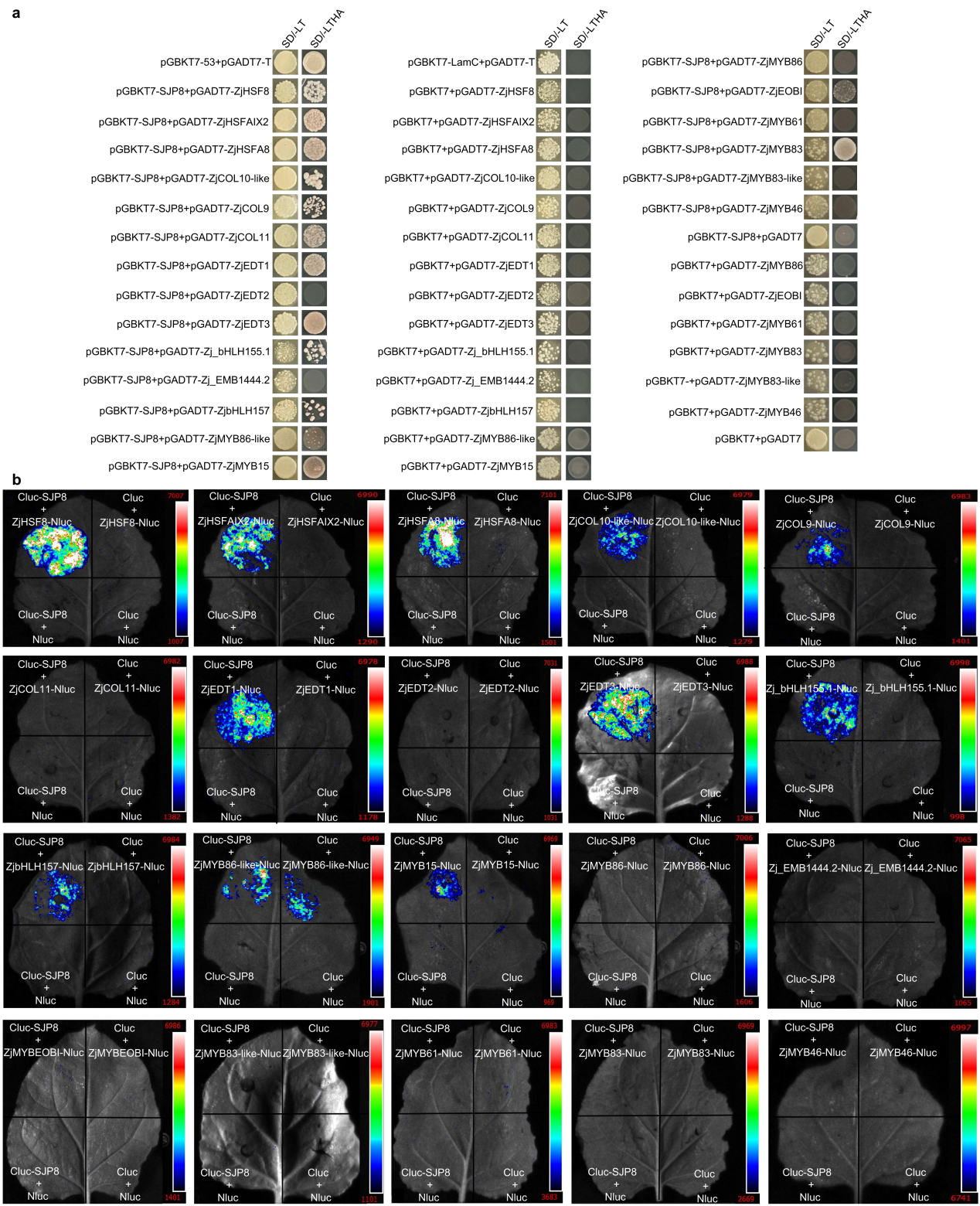


**Figure S12 |** Y2H and Split‑LUC assays validating the interaction of SJP8 with jujube homologs of selected *A. thaliana* transcription factors. (a) Y2H assays testing the interaction of SJP8 with jujube homologs of AtHSFA1D, AtBBX27, AtEDT1, AtbHLH155, and AtMYB61. pGBKT7-53 + pGADT7-T served as positive controls, while pGBKT7-LamC + pGADT7-T, pGBKT7 + pGADT7, and empty vectors were used as negative controls. SD/‑LT indicates SD/‑Trp‑Leu, and SD/‑LTHA indicates SD/‑Trp‑Leu‑His‑Ade. (b) Split‑LUC assay confirming the interactions of SJP8.
