# Supplementary material for: Phytoplasma Effector SJP8 Suppresses Host Immunity by Promoting the Degradation of ZjMYB15 and ZjMYB86‐like to Perturb Jasmonic Acid and Hydrogen Peroxide Homeostasis in Jujube
Source: Mol Plant Pathol. 2026 Jul 10;27(7):e70315. doi: 10.1111/mpp.70315 (PMC13351939; doi:10.1111/mpp.70315)
Supplement: Supplementary file 14 — Figure S14: Interaction between SJP8 deletion mutants and ZjMYB15 and ZjMYB86‐like. [file MPP-27-e70315-s006.docx]

**Figure S14 |** Interaction between SJP8 deletion mutants and ZjMYB15 and ZjMYB86‑like. (a) Homology comparison of SAP11 and SJP8 protein sequences using MEGA11. A single black line indicates the signal peptide, double black lines indicate the nuclear localization signal (NLS), and double dashed lines indicate the CC domain. (b) Split‑LUC assay confirming the interaction between ZjMYB15 and SJP8 variants (SJP8△N, SJP8△C, SJP8△C△cc). Cluc and Nluc empty vectors served as controls. (c) Y2H assay validating the protein interaction binding site based on the deletion mutant constructs shown in Fig. 8E. pGBKT7‑53 + pGADT7‑T served as positive controls; pGBKT7‑LamC + pGADT7‑T, pGBKT7, and pGADT7 were used as negative controls. S, serine; T, threonine; E, glutamic acid; D, aspartic acid; K, lysine; R, arginine. SD/‑LT: SD/‑Trp‑Leu; SD/‑LTHA: SD/‑Trp‑Leu‑His‑Ade. (d) Split‑LUC assay confirming the interaction between ZjMYB86‑like and the same SJP8 variants. Cluc and Nluc empty vectors served as controls.
