# Supplementary material for: Phytoplasma Effector SJP8 Suppresses Host Immunity by Promoting the Degradation of ZjMYB15 and ZjMYB86‐like to Perturb Jasmonic Acid and Hydrogen Peroxide Homeostasis in Jujube
Source: Mol Plant Pathol. 2026 Jul 10;27(7):e70315. doi: 10.1111/mpp.70315 (PMC13351939; doi:10.1111/mpp.70315)
Supplement: Supplementary file 15 — Figure S15: Expression analysis of jasmonic acid‐ and H2O2‐related genes Jingzao39 and Nicotiana benthamiana leaves transiently overexpressing SJP8 and its deletion mutants. [file MPP-27-e70315-s020.docx]

**Figure S15 |** Expression analysis of JA- and H₂O₂-related genes ‘Jingzao39’ and *N. benthamiana* leaves transiently overexpressing SJP8 and its deletion mutants. (a) Relative expression levels of H₂O₂ production, scavenging, and signal transduction genes in ‘Jingzao 39’ leaves at 72 hpi, determined by qRT-PCR. *ZjActin* was used as an internal reference gene. (b) Western blot detection of SJP8 and its deletion mutants in *N. benthamiana* leaves at 72 hpi. The Rubisco large subunit, visualized by Coomassie blue staining, served as a loading control. Molecular weight markers (kDa) are indicated on the left. (c) Relative expression levels of dwarfing‑related genes in *N. benthamiana* stems at 72 hpi, determined by qRT‑PCR. (d) Relative expression levels of chlorosis‑related genes in *N. benthamiana* leaves at 72 hpi, determined by qRT-PCR. (e-g) Relative expression levels of JA biosynthesis (e), metabolism (f), and signal transduction (g) genes in *N. benthamiana* leaves at 72 hpi, determined by qRT-PCR. (h) Relative expression levels of H₂O₂ production, scavenging, and signal transduction genes in *N. benthamiana* leaves at 72 hpi, determined by qRT-PCR. For panels (c-h), *NbActin* was used as an internal reference gene. For panels (a) and (e-h), statistical analysis was performed using one-way ANOVA with Tukey’s test. Error bars represent the SD of three technical replicates. Significance levels are indicated as follows: ns, not significant *p* > 0.05, **p* < 0.05, ***p* < 0.01, ****p* < 0.001, *****p* < 0.0001. All experiments were repeated three times with consistent results.
