# Supplementary material for: Phytoplasma Effector SJP8 Suppresses Host Immunity by Promoting the Degradation of ZjMYB15 and ZjMYB86‐like to Perturb Jasmonic Acid and Hydrogen Peroxide Homeostasis in Jujube
Source: Mol Plant Pathol. 2026 Jul 10;27(7):e70315. doi: 10.1111/mpp.70315 (PMC13351939; doi:10.1111/mpp.70315)
Supplement: Supplementary file 16 — Figure S16: Verification of ZjMYB15 and ZjMYB86‐like overexpression and RNAi transgenic Jingzao 39 lines. [file MPP-27-e70315-s038.docx]

**Figure S16 |** Verification of *ZjMYB15* and ZjMYB86-like overexpression and RNAi transgenic ‘Jingzao 39’ lines. (a) QRT-PCR analysis of *ZjMYB15* and *ZjMYB86-like* expression in leaves of SJP8-overexpressing transgenic jujube plants. GFP-transgenic plants served as the control. (b-c) QRT-PCR analysis of *ZjMYB15* and *ZjMYB86-like* expression in leaves (b) and stems (c) of healthy and JWB-infected plants. Healthy jujube plants served as the control. (d) Subcellular localization of ZjMYB15 and ZjMYB86-like. An empty GFP vector was used as a control. The pBI121-*OsGRX20*-mCherry construct (mCherry fluorescence) served as a nuclear-cytoplasmic co-localization marker, as *OsGRX20* has been reported to localize to both the nucleus and cytoplasm (Ning et al., 2018). Scale bar = 25 µm. (e) PCR-based DNA-level identification of transgenic lines. M: 2000 bp DNA marker; H_2_O: negative control. P1: pBI121-*ZjMYB15*-GFP (overexpression construct); OE-1, OE-2, OE-3: positive overexpression lines for *ZjMYB15*. P2: pFGC5941-*ZjMYB15* (RNAi construct); RNAi-1, RNAi-2, RNAi-3: positive RNAi lines for *ZjMYB15*. P3: pBI121-*ZjMYB86-like*-GFP (overexpression construct); OE-1, OE-2, OE-3: positive overexpression lines for *ZjMYB86-like*. P4: pFGC5941-*ZjMYB86-like* (RNAi construct); RNAi-1, RNAi-2, RNAi-3: positive RNAi lines for *ZjMYB86-like*. P1-P4 served as positive controls. Black arrows indicate the expected sizes of the PCR products. (f) QRT-PCR validation of *ZjMYB15* and *ZjMYB86-like* expression in the overexpression and RNAi transgenic lines. For panels (a-c) and f, *ZjActin* was used as an internal reference gene. Statistical analysis was performed using one-way ANOVA with Tukey’s test. Error bars represent the SD of three technical replicates. Significance levels are indicated as follows: **p* < 0.05, ***p* < 0.01, ****p* < 0.001, *****p* < 0.0001. All experiments were repeated three times with consistent results.
