# Supplementary material for: Phytoplasma Effector SJP8 Suppresses Host Immunity by Promoting the Degradation of ZjMYB15 and ZjMYB86‐like to Perturb Jasmonic Acid and Hydrogen Peroxide Homeostasis in Jujube
Source: Mol Plant Pathol. 2026 Jul 10;27(7):e70315. doi: 10.1111/mpp.70315 (PMC13351939; doi:10.1111/mpp.70315)
Supplement: Supplementary file 17 — Figure S17: ZjMYB15 and ZjMYB86‐like positively regulate shoot growth in Jingzao 39. [file MPP-27-e70315-s031.docx]

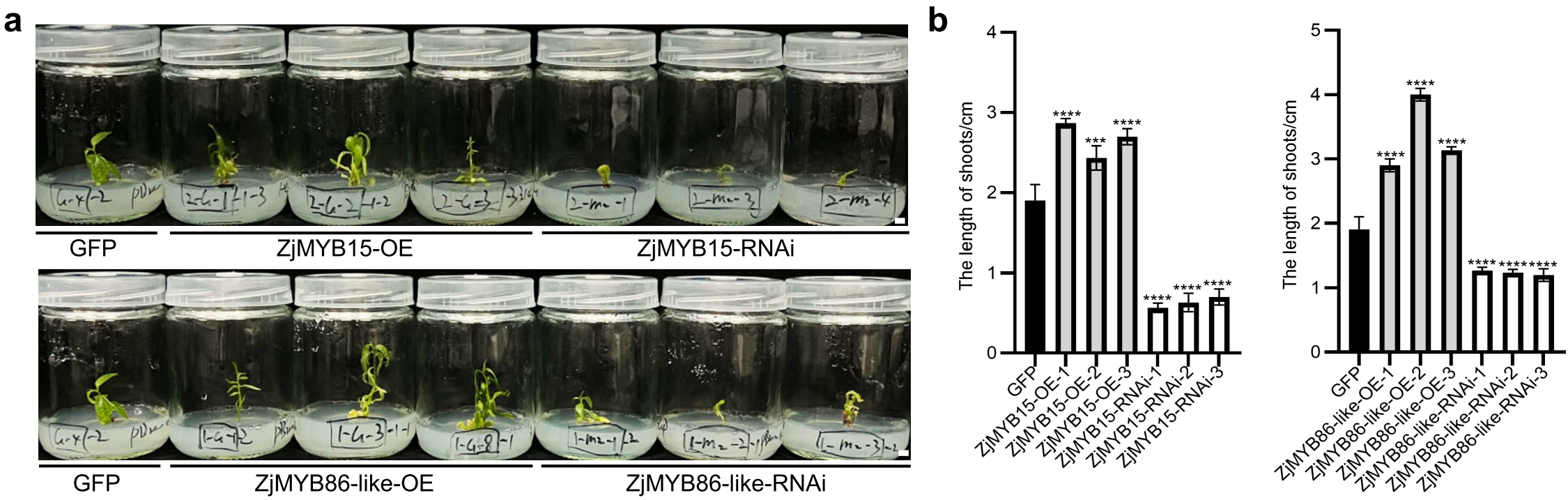


**Figure S17** | ZjMYB15 and ZjMYB86-like positively regulate shoot growth in ‘Jingzao 39’. (a) Phenotypes of transgenic ‘Jingzao 39’ lines overexpressing (OE) or RNAi-silencing (RNAi) ZjMYB15 or ZjMYB86-like after three weeks of subculture. Three independent transgenic lines were analyzed per construct. (b) Quantification of shoot length for the plants shown in (a). Data are presented as mean ± SD (n = 3). Statistical significance was determined by one-way ANOVA (****p* < 0.001, *****p* < 0.0001).
