# Supplementary material for: Phytoplasma Effector SJP8 Suppresses Host Immunity by Promoting the Degradation of ZjMYB15 and ZjMYB86‐like to Perturb Jasmonic Acid and Hydrogen Peroxide Homeostasis in Jujube
Source: Mol Plant Pathol. 2026 Jul 10;27(7):e70315. doi: 10.1111/mpp.70315 (PMC13351939; doi:10.1111/mpp.70315)
Supplement: Supplementary file 18 — Figure S18: Expression of ZjLOX2 and ZjCuAOβ in ZjMYB15 and ZjMYB86‐like transgenic lines. [file MPP-27-e70315-s035.docx]

**Figure S18** | Expression of *ZjLOX2* and *ZjCuAOβ* in *ZjMYB15* and *ZjMYB86-like* transgenic lines. (a) QRT‑PCR analysis of the JA-related gene *ZjLOX2* in overexpression (OE) and RNAi lines. (b) QRT‑PCR analysis of the H₂O₂-related gene *ZjCuAOβ* in the same lines. *ZjActin* was used as an internal reference. Data are presented as mean ± SD (n = 3). Statistical significance was determined by one-way ANOVA (**p* < 0.05, ***p* < 0.01, ****p* < 0.001, *****p* < 0.0001).
