# Supplementary material for: Phytoplasma Effector SJP8 Suppresses Host Immunity by Promoting the Degradation of ZjMYB15 and ZjMYB86‐like to Perturb Jasmonic Acid and Hydrogen Peroxide Homeostasis in Jujube
Source: Mol Plant Pathol. 2026 Jul 10;27(7):e70315. doi: 10.1111/mpp.70315 (PMC13351939; doi:10.1111/mpp.70315)
Supplement: Supplementary file 19 — Figure S19: ZjLOX2 expression in ZjMYB15 and ZjMYB86‐like transgenic lines in response to Pseudomonas syringae pv. tomato DC3000 infection. [file MPP-27-e70315-s030.docx]

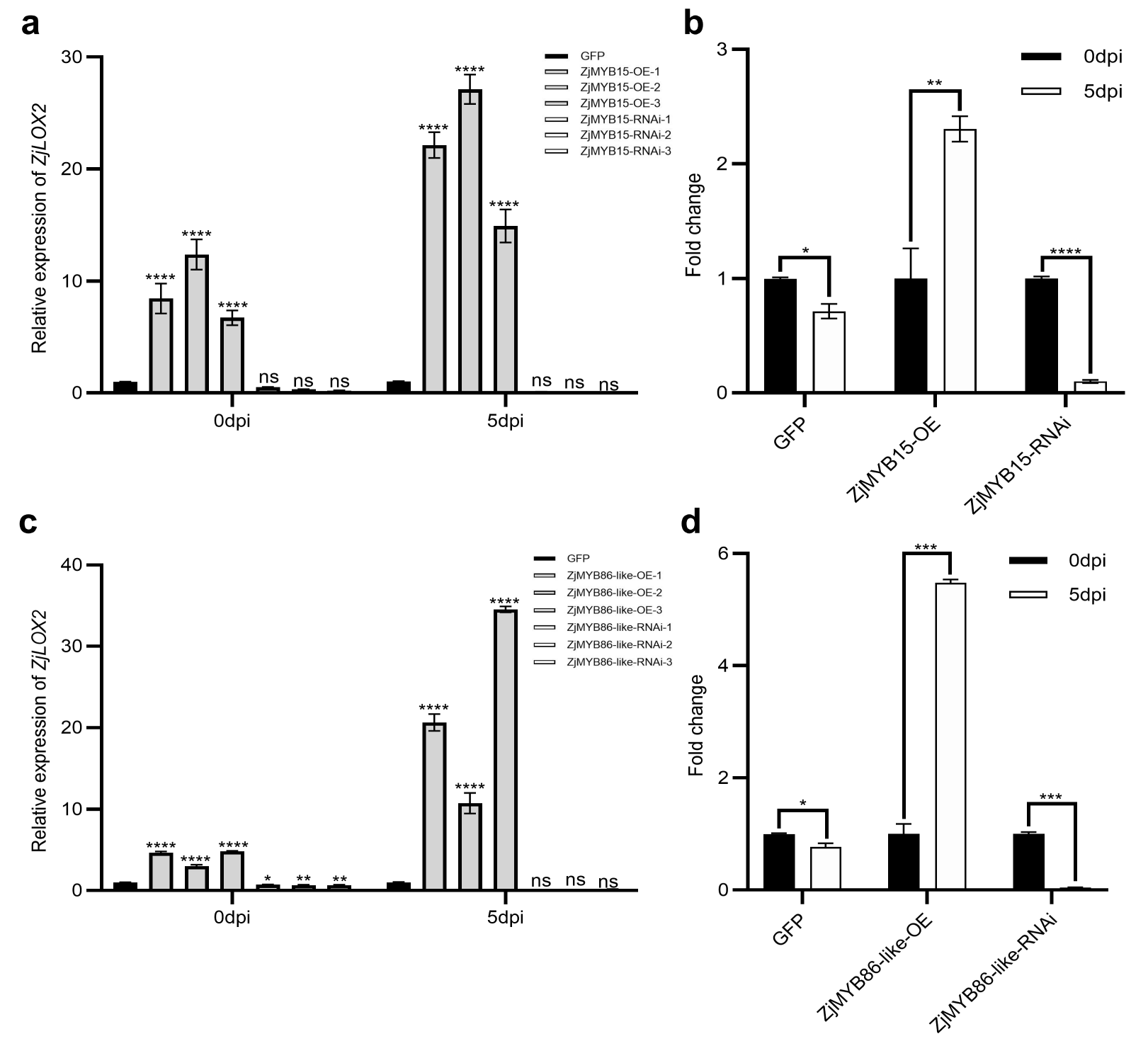


**Figure S19** | *ZjLOX2* expression in *ZjMYB15* and *ZjMYB86-like* transgenic lines in response to *Pst* DC3000 infection. (a) QRT-PCR analysis of *ZjLOX2* expression in leaves of *ZjMYB15*-overexpressing (OE) and RNAi lines at 5 dpi with *Pst* DC3000. Leaves at 0 dpi served as the baseline control. (b) Fold change in *ZjLOX2* expression at 5 dpi relative to 0 dpi for the lines shown in (a). (c) QRT-PCR analysis of *ZjLOX2* expression in *ZjMYB86-like*-OE and RNAi lines under the same conditions. (d) Fold change in *ZjLOX2* expression for the lines shown in (c). GFP-expressing plants served as controls in all panels. *ZjActin* was used as an internal reference. Data in (a) and (c) are presented as mean ± SD of three technical replicates. Statistical significance was determined by one-way ANOVA for (a) and (c) and by Student’s t-test for (b) and (d). Significance levels are indicated as follows: ns, not significant (*p* > 0.05); **p* < 0.05, ***p* < 0.01, ***p < 0.001, *****p* < 0.0001. All experiments were repeated three times independently with consistent results.
