# Supplementary material for: Phytoplasma Effector SJP8 Suppresses Host Immunity by Promoting the Degradation of ZjMYB15 and ZjMYB86‐like to Perturb Jasmonic Acid and Hydrogen Peroxide Homeostasis in Jujube
Source: Mol Plant Pathol. 2026 Jul 10;27(7):e70315. doi: 10.1111/mpp.70315 (PMC13351939; doi:10.1111/mpp.70315)
Supplement: Supplementary file 20 — Figure S20: Quantification of ZjMYB15 and ZjMYB86‐like protein levels upon proteasome inhibition. [file MPP-27-e70315-s009.docx]

**Figure S20** | Quantification of ZjMYB15 and ZjMYB86-like protein levels upon proteasome inhibition. (a) Quantification of ZjMYB15-FLAG band intensities from leaves co-expressing SJP8 and ZjMYB15, treated with DMSO (vehicle control), MG132, or Bortezomib. The GFP + ZjMYB15 and SJP8-GFP + ZjMYB15 combinations treated with DMSO served as controls. (b) Quantification of ZjMYB86-like-FLAG band intensities under the same experimental conditions, with the corresponding DMSO-treated groups as controls. Band intensities were normalized to Actin, and the value of the respective negative control was set to 1.00. Data are presented as mean ± SD (n = 3). Statistical significance was determined by one-way ANOVA (**p* < 0.05, *****p* < 0.0001).
