# Supplementary material for: Phytoplasma Effector SJP8 Suppresses Host Immunity by Promoting the Degradation of ZjMYB15 and ZjMYB86‐like to Perturb Jasmonic Acid and Hydrogen Peroxide Homeostasis in Jujube
Source: Mol Plant Pathol. 2026 Jul 10;27(7):e70315. doi: 10.1111/mpp.70315 (PMC13351939; doi:10.1111/mpp.70315)
Supplement: Supplementary file 21 — Figure S21: Degradation curves of ZjMYB15‐FLAG and ZjMYB86‐like‐FLAG. [file MPP-27-e70315-s013.docx]

**Figure S21** | Degradation curves of ZjMYB15-FLAG and ZjMYB86-like-FLAG. (a) Degradation curve of ZjMYB15-FLAG. (b) Degradation curve of ZjMYB86-like-FLAG. Band intensities were normalized to Actin and expressed as a percentage of the 0 h time point. Blue lines represent the CHX-EV (GFP) and CHX-SJP8 groups. The pink line indicates the CHX + MG132-treated SJP8 + target protein group, and the black line denotes the CHX + MG132-treated EV (GFP) + target protein control. Statistical significance of differences in remaining protein levels at 4 h was assessed by one-way ANOVA (*****p* < 0.0001). Data are presented as mean ± SD (n = 3)
