# Supplementary material for: Phytoplasma Effector SJP8 Suppresses Host Immunity by Promoting the Degradation of ZjMYB15 and ZjMYB86‐like to Perturb Jasmonic Acid and Hydrogen Peroxide Homeostasis in Jujube
Source: Mol Plant Pathol. 2026 Jul 10;27(7):e70315. doi: 10.1111/mpp.70315 (PMC13351939; doi:10.1111/mpp.70315)
Supplement: Supplementary file 22 — Figure S22: Subcellular localisation of ZjPOD43 and ZjJAIPHX1 in Nicotiana benthamiana. [file MPP-27-e70315-s044.docx]

**Figure S22 |** Subcellular localization of ZjPOD43 and ZjJAIPHX1 in *N. benthamiana*. (a) Subcellular localization under normal conditions. (b) Plasmolysis experiments verifying subcellular localization. An empty GFP vector served as a control. The pBI121-*OsGRX20*-mCherry construct (mCherry fluorescence) served as a nuclear-cytoplasmic co-localization marker, as *OsGRX20* has been reported to localize to both the nucleus and cytoplasm (Ning et al., 2018). Scale bar = 25 µm.
