# Supplementary material for: Phytoplasma Effector SJP8 Suppresses Host Immunity by Promoting the Degradation of ZjMYB15 and ZjMYB86‐like to Perturb Jasmonic Acid and Hydrogen Peroxide Homeostasis in Jujube
Source: Mol Plant Pathol. 2026 Jul 10;27(7):e70315. doi: 10.1111/mpp.70315 (PMC13351939; doi:10.1111/mpp.70315)
Supplement: Supplementary file 23 — Figure S23: ZjMYB15 and ZjMYB86‐like activate transcription of ZjJAIPHX1 and ZjPOD43. [file MPP-27-e70315-s027.docx]

**Figure S23 |** ZjMYB15 and ZjMYB86‑like activate transcription of *ZjJAIPHX1* and *ZjPOD43*. (a) Dual‑luciferase reporter assay showing activation of the *ZjJAIPHX1* and *ZjPOD43* promoters by ZjMYB15. The injection layout and measured luciferase activities are indicated. pGreenII 62‑SK and pGreenII 0800 were used as negative controls. “P” indicates the promoter region; “1‑P” corresponds to *proZjJAIPHX1*, and “2‑P” corresponds to *proZjPOD43*. (b) Dual‑luciferase reporter assay showing activation of the same promoters by ZjMYB86‑like, with the same controls and labeling as in (a). (c) QRT‑PCR analysis of *ZjJAIPHX1* and *ZjPOD43* expression in ZjMYB15‑overexpression and RNAi transgenic jujube lines. (d) QRT‑PCR analysis of *ZjJAIPHX1* and *ZjPOD43* expression in ZjMYB86‑like‑overexpression and RNAi transgenic jujube lines. *ZjActin* was used as an internal reference gene. For panels (a)-(d), statistical significance was assessed using one‑way ANOVA. Error bars represent the SD of three technical replicates. Significance levels are indicated as follows: **p* < 0.05, ***p* < 0.01, ****p* < 0.001, *****p* < 0.0001.
