# Supplementary material for: Phytoplasma Effector SJP8 Suppresses Host Immunity by Promoting the Degradation of ZjMYB15 and ZjMYB86‐like to Perturb Jasmonic Acid and Hydrogen Peroxide Homeostasis in Jujube
Source: Mol Plant Pathol. 2026 Jul 10;27(7):e70315. doi: 10.1111/mpp.70315 (PMC13351939; doi:10.1111/mpp.70315)
Supplement: Supplementary file 24 — Figure S24: Reverse transcription‐quantitative PCR analysis of JAIPHX1 and POD43 expression in healthy, infected and SJP8‐overexpressing transgenic plants. [file MPP-27-e70315-s022.docx]

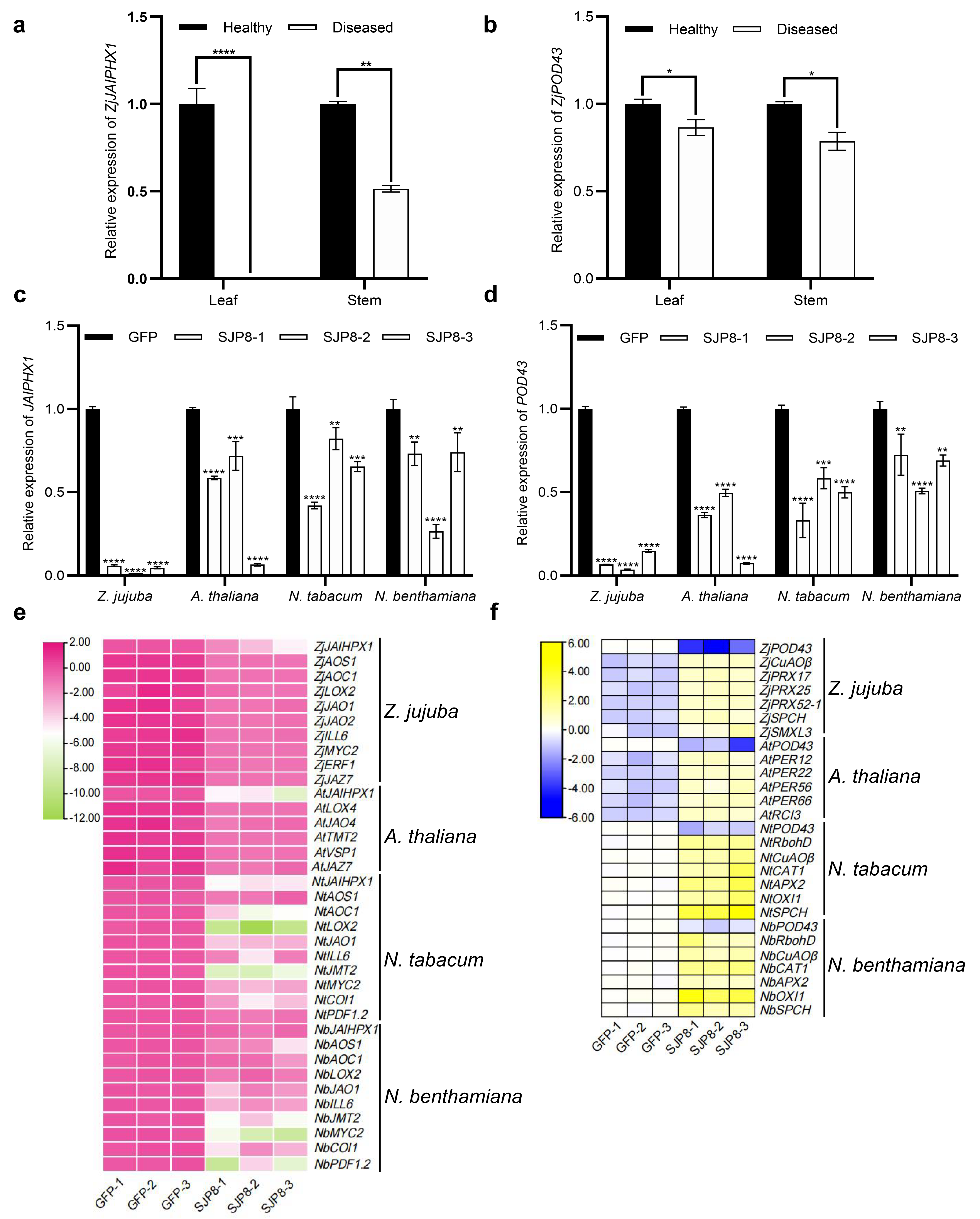


**Figure S24 |** QRT‑PCR analysis of *JAIPHX1* and *POD43* expression in healthy, infected, and SJP8‑overexpressing transgenic plants. (a) Expression of *ZjJAIPHX1* in leaves and stems of healthy and JWB‑infected plants. Healthy plants served as the controls. (b) Expression of *ZjPOD43* in leaves and stems of healthy and JWB‑infected plants. Healthy plants served as controls. (c) Expression of *JAIPHX1* homologs in leaves of SJP8‑overexpressing *Z.jujuba* (‘Jingzao39), *A. thaliana*, *N. tabacum*, and *N. benthamiana* plants. GFP‑expressing empty vector served as the control. (d) Expression of *POD43* homologs in leaves of SJP8‑overexpressing *Z.jujuba* (‘Jingzao39), *A. thaliana*, *N. tabacum*, and *N. benthamiana* plants. GFP‑expressing empty vector served as the control. For panels (a-d), *ZjActin* was used as an internal reference gene. Statistical analysis was performed using one‑way ANOVA with Tukey’s test. Error bars represent the SD of three technical replicates. Significance was defined as *p* < 0.05. All experiments were repeated three times with consistent results. (e) Clustering analysis of *JAIHPX1* homologs together with relevant marker genes in SJP8-transgenic plants. (f) Clustering analysis of *POD43* homologs together with relevant marker genes in SJP8-transgenic plants. Significance levels are indicated as follows: **p* < 0.05, ***p* < 0.01, ****p* < 0.001, *****p* < 0.0001.
