# Supplementary material for: Phytoplasma Effector SJP8 Suppresses Host Immunity by Promoting the Degradation of ZjMYB15 and ZjMYB86‐like to Perturb Jasmonic Acid and Hydrogen Peroxide Homeostasis in Jujube
Source: Mol Plant Pathol. 2026 Jul 10;27(7):e70315. doi: 10.1111/mpp.70315 (PMC13351939; doi:10.1111/mpp.70315)
Supplement: Supplementary file 25 — Figure S25: Conservation analysis of the ZjMYB15‐binding motif. [file MPP-27-e70315-s005.docx]

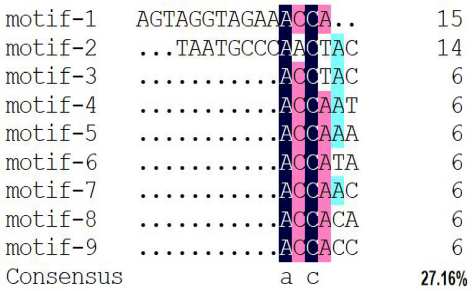


**Figure S25 |** Conservation analysis of the ZjMYB15-binding motif. The ZjMYB15-binding motifs identified by DAP-seq (motif-1: AGTAGGTAGAAACCA; motif-2: TAATGCCCAACTACT) were aligned with known AtMYB61-binding motifs (motif-3 to motif-9) using DNAMAN (Prouse et al., 2013). The alignment reveals an AC-rich core and a sequence identity of 27.16%.
